# TIA1 regulates the generation and response to toxic tau oligomers

**DOI:** 10.1007/s00401-018-1937-5

**Published:** 2018-11-21

**Authors:** Lulu Jiang, Peter E. A. Ash, Brandon F. Maziuk, Heather I. Ballance, Samantha Boudeau, Ali Al Abdullatif, Marcello Orlando, Leonard Petrucelli, Tsuneya Ikezu, Benjamin Wolozin

**Affiliations:** 1 Department of Pharmacology and Experimental Therapeutics, Boston University, School of Medicine, Boston, MA 02118 USA; 2 Neuroscience Division, Mayo Clinic, Jacksonville, FL 32224 USA; 3 Department of Neurology, Boston University School of Medicine, Boston, MA 02118 USA

**Keywords:** Tau propagation, Tauopathy, Neurodegeneration, RNA binding proteins, TIA1, Tau oligomers, Tau fibrils, Stress granules, Neuropathology

## Abstract

RNA binding proteins (RBPs) are strongly linked to the pathophysiology of motor neuron diseases. Recent studies show that RBPs, such as TIA1, also contribute to the pathophysiology of tauopathy. RBPs co-localize with tau pathology, and reduction of TIA1 protects against tau-mediated neurodegeneration. The mechanism through which TIA1 reduction protects against tauopathy, and whether TIA1 modulates the propagation of tau, are unknown. Previous studies indicate that the protective effect of TIA1 depletion correlates with both the reduction of oligomeric tau and the reduction of pathological TIA1 positive tau inclusions. In the current report, we used a novel tau propagation approach to test whether TIA1 is required for producing toxic tau oligomers and whether TIA1 reduction would provide protection against the spread of these oligomers. The approach used young PS19 P301S tau mice at an age at which neurodegeneration would normally not yet occur and seeding oligomeric or fibrillar tau by injection into hippocampal CA1 region. We find that propagation of exogenous tau oligomers (but not fibrils) across the brain drives neurodegeneration in this model. We demonstrate that TIA1 reduction essentially brackets the pathophysiology of tau, being required for the production of tau oligomers, as well as regulating the response of neurons to propagated toxic tau oligomers. These results indicate that RNA binding proteins modulate the pathophysiology of tau at multiple levels and provide insights into possible therapeutic approaches to reduce the spread of neurodegeneration in tauopathy.

**Electronic supplementary material:**

The online version of this article (10.1007/s00401-018-1937-5) contains supplementary material, which is available to authorized users.

## Introduction

RNA binding proteins (RBPs) are strongly implicated in neurodegeneration. Mutations in RBPs are associated with motor neuron diseases and form a strong component of the pathology of these diseases. Recent studies demonstrate that RBPs, stress granules (SGs) and the translational stress response are also key pathways mediating the pathophysiology of tauopathy [8, 9, 43]. SGs are membraneless organelles composed of RNA binding proteins (RBPs) and mRNA, which regulate the translational stress response [4, 28]. Somatodendritic mislocalization of tau stimulates the formation of SGs containing TIA1, which is a RBP that nucleates SGs and is genetically linked to neurodegeneration and myopathy [18, 23, 27]. Prolonged association of tau with SGs appears to stimulate tau aggregation [2, 43]. The requirement for TIA1 in the pathophysiology of tauopathy was demonstrated by recent findings that TIA1 reduction prolongs lifespan and provides neuroprotection in the PS19 P301S tau mouse model, in a manner that reduced oligomeric tau but increased fibrillary tau [2]. The surprising effects of TIA1 on oligomeric tau raises fundamental mechanistic questions: does TIA1 provide neuroprotection in a cell autonomous manner by modulating production of toxic tau oligomers or does it act in a cell dependent manner by modulating the response of neurons to tau oligomers?

The current study uses tau seeding and propagation models to investigate the mechanisms through which TIA1 impacts on tauopathy. In tauopathies, misfolding or aggregated tau forms oligomers and fibrils [47]. Emerging evidence has shown that tau pathology expands through a seeding mechanism that produces intercellular propagation and spread of pathology across brain areas [6, 39]. Uptake and cell to cell transfer of extracellular tau aggregates have been demonstrated in cell culture and in vivo [10, 15, 37, 40]. Studies of tau seeding and propagation offer an extraordinary model for mechanistic studies by enabling the study of generation of tau oligomers and the toxicity of tau oligomers separately. These studies indicate that the pattern of tau pathology induced by tau propagation differs depending on the type of tau species contained, such as oligomeric or fibrillar tau [19, 21, 22, 50]. Indeed, although multiple studies examine the distribution of tau pathology, few studies have examined the mechanisms through which propagated tau cause degeneration [16].

The current study investigates the activities of tau species in propagating tau pathology and neurodegeneration and demonstrates a key role for TIA1 and SGs in neurodegeneration mediated by extracellular, propagated tau oligomers. The critical role of TIA1 enabled further investigation into the pathophysiological mechanisms through which TIA1 contributes to tauopathy. This study begins by comparing the effects in vivo and in vitro of different tau species extracted from P301S tau mouse brains and demonstrates that tau oligomer and fibrillar fractions both propagate tau pathology in vitro and in vivo, but exhibit striking differences in the neurodegenerative outcomes. The oligomeric tau fraction induced tau pathology in neuronal soma that co-localized with SG markers and elicited profound neurodegeneration. In contrast, over the 3-month period of this study, the fibrillar tau fractions induced tau inclusions predominantly in synapses and dendrites, but showed no association with SG markers, nor did they elicit neurodegeneration. Next, we further use this model for mechanistic studies. We show that TIA1 reduction elicited neuroprotection through both cell autonomous and cell non-autonomous mechanisms. Mice with reduced TIA1 produce less toxic tau oligomers and also exhibit reduced vulnerability to toxic tau oligomers generated from elderly P301S MAPT mice. These results demonstrate that oligomeric tau propagates toxic tau pathology through a mechanism mediated by TIA1 and pathological SGs, suggesting a broad role for SGs in the mechanisms of tau-mediated neurodegeneration.

## Materials and methods

### Animals

Use of all animals was approved by the Boston University Institutional and Animal Care and Use Committee. All animals were housed in IACUC-approved vivariums at Boston University School of Medicine. *Tia1*^−/−^ mice (B6.129S2(C)-*Tia1*tm1Andp/J) were generated in and obtained from Anderson lab in Harvard University, Dana Farber Cancer Institute [34]; these mice had previously been backcrossed for 10 + generations to a C57BL/6 genetic background. PS19 mice overexpressing human P301S Tau (B6;C3-Tg(Prnp-MAPT*P301S)PS19Vle/J, stock#008,169) and C57BL/6 J mice (stock#000664) were purchased from Jackson Laboratories [51]. Generation of *P301S*::*Tia1*^+*/*+^ and *P301S*::*Tia1*^+*/*−^ mice were as described previously [2]. Briefly, PS19 mice were bred with *Tia1*^−/−^ to produce F1 generation of *P301S*^+*/*−^::*Tia1*^+/−^ mice. Then *P301S *± ::*Tia1*^+/−^ mice were set as breeders for producing *P301S*^+*/*−^::*Tia1*^+/+^, *P301S*^+*/*−^*::Tia1*^+/−^ and *P301S*^−*/*−^::*Tia1*^+*/*+^ mice used in this study. Equal numbers of male and female mice were used in all experimental comparisons; no statistically significant differences with respect to sex were observed in this study (data not shown). Littermates of the same sex were randomly assigned to experimental groups. Mice underwent stereotaxic injection at 3 months and then were killed after three more months, at 6 months of age. Timed pregnant C57BL/6 were purchased from Charles River laboratories and delivered at E-14. Primary hippocampal cultures were generated from postnatal P0 pups.

### Primary hippocampal neuronal cultures

P0 neonatal C57BL/6 mouse pups were used to generate the neuronal cultures. For hippocampus dissection, the P0 pups were anesthetized via hypothermia by wrapping in gauze and placing in aluminum foil pouch on ice. The hippocampi were removed and treated with trypsin and DNase by immersion in HBSS dissection buffer with 5 ml 0.25% Trypsin–EDTA supplemented with 150 μL DNase. The samples were incubated 15 min in a 37 °C water bath for 15 min, washed three times with HBSS dissection buffer, spin down in 2000 g for 2 min at room temperature. The resulting cells were resuspended in 2 ml plating medium (MEM Gibco #11090, 2.5% FBS, 1 × Penicillin/streptomycin, l-glutamine, 0.6% d-glucose), gently triturated, passed through a 70-μm cell strainer and then the resulting cell count quantified. Plating was done with 60,000 cells/coverslip in 80 µl medium (7.5 × 10^5^ cells/ml) for a 24-well plate; after 30 min 1 ml of feeding medium (Neurobasal media, 1 × B27 supplement, 1 × Penicillin/streptomycin, 1 × l-glutamine) was added to each well. The cultures were maintained at 37 °C in the incubator with 5% CO_2_ and 95% air.

The cover slips used for plating were prepared as follows: Sterilized 12 mm coverslips were placed into each well of 24-well plate and then coated with 80 µl of 1 mg/ml poly-d-lysine (only on the coverslip) for 1 h at room temperature in the culture hood. The plates were washed three times with sterile biology grade water and dried in hood overnight covered in foil.

### S1p and P3 fractions extraction from aged PS19 brain tissue

Frozen hippocampus and cortical tissues of 9-month-old PS19 mice were weighed (100 mg–250 mg) and put in Beckman Centrifuge Tube, polycarbonate thick wall (cat # 362305). A 10 × volume of homogenization buffer was used to homogenize brain tissue with Hsaio TBS buffer (50 mM Tris, pH 8.0, 274 mM NaCl, 5 mM KCl) supplemented with protease and phosphatase inhibitor cocktails (Roche, cat#05892791001 and cat#04906837001), as described previously [2].

#### Generation of the S1p fraction

The homogenate was centrifuged at 48,300*g* for 20 min at 4 °C. The supernatant is designated as the S1 (TBS-soluble) fraction. The supernatant (S1) fraction was centrifuged a second time at 186,340*g* at 4 °C for 40 min. The TBS-extractable pellet (S1p) fraction was resuspended in a 4x volume of TE buffer relative to the starting weight of the tissue homogenate, aliquotted and frozen.

#### Generation of the P3 fraction

The pellet (P1) was homogenized with buffer B (10 mM Tris, pH 7.4, 800 mM NaCl, 10% sucrose, 1 mM EGTA, 1 mM PMSF), ~ 5x volume of wet weight of the original tissue. This homogenate was centrifuged homogenate at 29,800*g* for 20 min at 4 °C. The resulting supernatant (S2) was transferred to a new Beckman polycarbonate thick-walled tube and with 1% Sarkosyl by rotating in the bench top thermomixer at 37 °C for 1 h. This sample was centrifuged at 186,340*g* for 1 h at 4 °C. The sarkosyl-insoluble pellet (P3) was resuspended with 50 µl TE buffer (10 mM Tris, 1 mM EDTA, pH 8.0).

The molecular weight of tau in the S1p and P3 fractions was documented by native page gel electrophoresis, and the concentration of total tau was measured by immunoblot using 3–12% reducing SDS-PAGE gel by comparison to a gradient concentrations of recombinant tau ladders, using the tau-5 antibody (detecting total tau) by immunoblot (supplemental Fig. 1). All the fractions were then normalized and divided into fractions of 20 µg/ml tau for storage and future use.

### Cell transduction

For cell transduction, AAV were added between days 2 and 5 to over-express or knock down target protein. Briefly, at day 2, neurons were transduced with AAV1 vectors of human 4R0 N WT tau or P301L tau at MOI 200. At day 5, neurons were transduced with AAV9-shctrl or shTIA1 virus (MOI 200). The conditioned culture medium was replaced 1/2 volume with fresh feeding media every 3–4 days for cell maintenance until the cells were ready to use for experiment on day-14 to day-21.

### Treatment of neuronal cultures with S1p and P3 fractions

S1p and P3 stock solution (20 mg/ml) were diluted in 1 ml feeding medium for each well in 24-well plates and added into the cells by completely replacing the old medium. By completely replacing the medium, the cells were starving for neuronal nutritional factors and more functional for neuronal activities. Then the supernatant was collected and cells were fixed by 4% PFA for being frozen in a time series (1 h, 2 h, 4 h, 24 h, 96 h) for further analysis.

### LDH assay

50 µl supernatant was collected as designed time point into a 96-well plate for lactate dehydrogenase (LDH) release assay as per manufacture’s protocol (Promega, cat# G1780). Briefly, 50 μl of the CytoTox 96^®^ Reagent was added to each sample aliquot. The plate was covered with foil to protect it from light and incubated for 30 min at room temperature on shaker. 50 μl of Stop Solution was added to each well of the 96-well plate and the absorbance recorded at 490 nm with the plate reader. Each experiment was repeated at least three times with triplicate wells each time.

### Immuno-depletion of tau from fractions

Tau aggregates in S1p fractions were eliminated from the fractions by a direct immuno-precipitation kit (Pierce, cat# 26148). Briefly, first tau-5 antibody was coupled to AminoLink plus Coupling Resin, and the fractions were pre-cleared using the Control Agarose Resin with all the materials provided by the kit. The sample was added to the antibody-coupled resin in the spin column and incubated in the column for overnight at 4 °C on a gentle rotator. The column was centrifuged, and the flow-through saved for further experimentation. After three washes with IP buffer, the spin column was placed into a new collection tube and tau plus antibodies were eluted from the resin. The eluate was analyzed for the presence of tau.

### Immuno-fluorescence staining of fixed primary culture

Cells on a 24-well cover slips were fixed with 0.5 ml 4% PFA/PBS for 15 min. The cells were washed three times in PBS, 5 min each wash. The cells were permeabilized in .0.5 ml PBS/0.1% Triton X-100 (PBST) for 15–30 min. Blocking was done in 0.5Ml of 5% BSA—5% donkey Serum in PBST for 1 h. Then the cells were incubated in primary antibodies diluted in 5% BSA/PBST at 4 °C overnight follwed by being washed 3 times in PBS-T, 10 min each, on the second day. The samples were incubated in 2° antibody diluted in 5% BSA/PBST, 2 h at RT. After incubation with the 2° antibody, the samples were incubated in DAPI diluted 1:10,000 in PBST (5 mg/ml stock solution) for 5 min after first wash. Then the samples were washed with 2x with PBST, and then once in PBS, 10 min each, after which the samples were mounted onto coverslips using Prolong Gold Antifade mounting media. The primary antibodies used in this study for ICC are as follows: NeuN (rabbit, Millipore, ABN78, 1: 500), MAP-2 (chicken, AVES, cat# MAP, 1: 250), MAP-2 (rabbit, Millipore, cat# AB5622, 1: 1000), V-5 (rabbit, Sigma-Aldrich, cat# V8137, 1:1000), CP-13 (mouse, provided by Peter Davies, 1:300), PHF1 (mouse, provided by Peter Davies, 1:300) [24], TIA1 (rabbit, abcam, cat# ab40693, specifically lot# GR3202325-1, 1:400). All the 2° antibodies were purchased from Thermo Fisher Scientific made in donkey and used for 1:800 dilution in staining. Images were captured by Zeiss AxioObserver Microscope or Carl Zeiss confocal LSM700.

### CA1 stereotactic injection

Littermates PS19 mice of *Tia1*^+*/*+^ or *Tia1*^+*/*−^ were stereotaxically injected with 2 µl saline, S1p (40 ng oligomeric tau) or P3 (40 ng fibrillary tau) fractions bilaterally in CA1 region at the age of 3 months. The coordinates of the injection site were 1.8 mm posterior and 1.5 mm lateral to bregma, 1.5 mm ventral to cortical surface [32]. The procedure of stereotaxic injection was performed with KOPF instruments supplemented with Neurostar software as described previously [3]. Briefly, mice were deeply anesthetized with isoflurane (4%, Abbot Laboratories) and placed in a stereotaxic frame. Anesthesia was kept constant with 1.5–2% isoflurane and oxygen pressure 6–8 kPa supplied per anesthesia nosepiece. After injecting one unit of meloxicam for every 10 g of body weight for each mouse under the skin as analgesia, the skull was exposed and perforated with a stereotaxic drill at the desired coordinates bilaterally. After the skull was drilled with a robot drill, the syringe was switched for saline or fraction injection. The speed for needle insertion into the brain was 0.2 mm/min and the speed for solution injection is 1 µl/min. The needle was left in place for 15 min after the injection volume was delivered, the syringe removed at a rate of 0.2 mm/min and the skin over the entry point sutured. The mice were transferred to a single cage with hot pad on the bottom. Meloxicam was injected every 12 h until the mice recovered completely.

### Immunohistochemistry

PS19 mice with *Tia1*^+*/*+^ or *Tia1*^+*/*−^ were killed at the age of 6 months (3 months after the S1p or P3 fraction injection). Briefly, mice were anesthetized with isoflurane and then the hearts perfused with 20 ml ice cold PBS for 5 min followed by perfusion with 20 ml ice-cold 4% PFA for 10 min. The mouse brains were dissected and placed in 4% PFA on ice for 2 h. Then the brains were washed with PBS and transferred into 30% sucrose/PBS until the brains sank to the bottom of the tube (about 48 h) and sectioned. The fixed brains were sliced into 30 µm coronal sections by cryostat and stored in 0.005% sodium azide/PBS solution at 4 °C for up to 3 months. For long-term storage, the sections were transferred into cryoprotectant solution (30% glycerol and 30% ethelyne glycol in PBS) and stored at −20 °C.

For immuno-labeling, the 30-µm free-floating sections with hippocampus or lateral entorhinal cortex (LEnt) were immunoblocked with 5% BSA and 5% goat serum in PBST (PBS/0.25% Triton X-100) for 30 min and then incubated with monoclonal mouse anti-NeuN antibody (Millipore, cat#MAB377, 1:1000 dilution) overnight at 4 °C. On the second day, sections were washed with PBST three times and then incubated with biotinylated goat anti-mouse IgG antibody (Vector Laboratories, cat# BA-9200) for 2 h RT. The antibody binding was visualized using a Vectastain ABC Kit (Vector Laboratories, cat# PK-6100) and diaminobenzidine substrate tablet (Sigma-Aldrich, cat# D4293-50SET) as described previously [20]. Images were captured by Zeiss Axio Observer Microscope.

### Immuno-fluorescence staining of fixed brain tissues

For immuno-labeling, selected sections of hippocampus from bregma − 1.8 and LEnt from bregma − 2.8 were washed in PBS for 10 min and then permeabilized in 0.5 ml PBS/0.25% Triton X-100 (PBST). Block tissues in blocking solution were supplemented with 5% BSA and 5% normal donkey serum in PBST, 1.5–2 h at RT. Then the brain sections were incubated in primary antibodies diluted in 5% BSA/PBST for overnight at 4 °C. On the second day, brain sections were washed 3 times in PBST, 15 min each. And then the brain sections were incubated in secondary antibodies (1:700 for Dylight-/Alexa-conjugated antibodies made in donkey purchased from Thermo Fisher Scientific) diluted in 5% BSA/PBST for 2 h at RT. For DAPI nuclei staining, 5mg/ml DAPI were diluted (1:10,000) in PBST and incubated with the sections for 15 min. After DAPI, the brain tissues were washed twice with PBST before the last wash with 1x PBS, 10 min each. At last, the brain sections were mounted onto microscope glass slides in Prolong gold antifade reagent. The primary antibodies used in this study for IHC are as follows: NeuN (chicken, Millipore, cat# ABN91), 1: 300; Tau-5 (mouse, provided by Nicholas Kanaan, MSU), 1:1000 [26]; MC1 (mouse, provided by Peter Davies, Northwell), 1:100; CP-13 (mouse, provided by Peter Davies, Northwell), 1:300; PHF1 (mouse, provided by Peter Davies, Northwell), 1:300 [48]; TOMA2 (mouse, provided by Rakez Kayed, UTMB Galveston), 1:200 [48]; TIA1 (rabbit, Abcam, cat# ab40693, specifically lot# GR3202325-1), 1:400. Images were captured by Carl Zeiss confocal LSM700.

### Thioflavin S staining in brain tissue

The fresh made Thioflavin S (ThioS) solution was prepared by dissolving 1 g of ThioS in 100 ml 80% ethanol and kept stirring overnight at 4 °C before filtered for final use. Selected 30 μm brain sections were mounted onto glass microscope slides and allowed to completely dry. Slides were washed sequentially in 70% and 80% ethanol, 1 min each, prior to incubating in ThioS/80% ethanol solution for 15 min. Sections were then sequentially washed in 80% and 70% ethanol, 1 min each, followed by two rinses in PBS. Slides were mounted in Prolong Gold antifade reagent and stored in the dark until imaging.

### Immunoblot

For the native page gel electrophoresis, S1p and P3 fractions were prepared with a Native PAGE sample prep kit (cat# BN2008) and then run on a Native PAGE Novex 3–12% bis–tris protein gels (Thermo Fisher Scientific, cat# BN1003BOX) with light blue Cathode Buffer. The molecular weight of tau aggregates in S1p and P3 fractions were then detected by Tau-13 antibody (provided by Nicholas Kanaan, 1:5000) immunoblot. For CP13 and MAP-2 detection in primary culture, cell lysate were collected from frozen cultures with RIPA lysis buffer. Reducing and non-reducing protein samples were separated by gel electrophoresis and transferred to 0.2 µm nitrocellulose membranes using the Bolt SDS-PAGE system (Life Technologies). Membranes were blocked in 5% nonfat dry milk (NFDM) in PBS supplemented with 0.025% Tween-20 (PBST) for 1 h RT, followed by incubation overnight at 4 °C in primary antibody diluted in 5% bovine serum albumin/PBST. Primary antibodies used were as follows: CP13 (1:500) anti-tau antibodies (generously provided by P. Davies), MAP-2 (rabbit, Millipore, cat# AB5622, 1: 5000), TIA1 (rabbit, abcam, cat# ab40693, 1:800). Membranes were then washed three times with PBST and incubated in HRP-conjugated secondary antibodies (Jackson ImmunoResearch) diluted in 1% BSA/PBST at RT for 1 h. After incubation in secondary antibody, membranes were washed three times in PBST and developed using SuperSignal West Pico Chemilluminescent ECL substrate (ThermoFisher Scientific, cat# 34080).

### Images analysis

The immuno-fluorescence stained NeuN-positive neurons in each image from primary hippocampal cultures were quantified by Image J with function of automatic cell counting. The dendritic length measurement of neurons in MAP-2 staining was quantified using ImageJ plug-ins NeuronJ to trace the MAP2 positive processes [38]. The staining intensity in immuno-fluorescence or DAB stained brain sections was measured by ImageJ; the NeuN-positive cells in LEnt sections were quantified by Image J automatica cell counting. Co-localization of TOMA-2 positive tau oligomers to TIA1 granules in neuronal soma in Fig. 3 was analyzed with z-stacks images and Pearson coefficient assay by FIJI (ImageJ) coloc2 plug-in. The quantification of cell numbers was done blindly by at least two investigators in the lab.

### Statistical analysis

Statistical analyses and figures artwork were performed using GraphPad Prism version 6.00 for Windows with two-sided *α* of 0.05. All group data are expressed as mean ± SEM. Colum means were compared using one-way ANOVA with treatment as the independent variable. And group means were compared using two-way ANOVA with factors on genotype and fractions treatment, respectively. When ANOVA showed a significant difference, pairwise comparisons between group means were examined by Tukey’s, Dunnett or uncorrected Fisher’s LSD multiple comparison test. Significance was defined when *p *< 0.05.

## Results

### Oligomeric tau fractions trigger neurotoxicity

Previous studies indicate that biochemical fractionation of tau pathology-affected tissues yields fractions that have predominantly oligomeric tau species (the S1p fraction) or fibrillar tau species (the P3 fraction) [5]; however, the relationship between these fractions and toxicity in propagation models has not been examined. Thus, we began the study by determining whether the oligomeric and fibrillar tau fractions differ in toxicity. To study the toxicity of different tau species, frozen brain tissue of 9-month-old PS19 P301S tau mice was homogenized and separated into S1p (the tau oligomer fraction) and P3 fractions (a biochemical fraction containing tau fibrils). Then the tau aggregates in S1p and P3 fractions were validated by immunoblot, which showed that the strongest tau signal in the S1p fraction was between 100 and 150 kD, whereas the strongest tau signal in the P3 fraction was more than 200 kD (supplemental Fig. 1a–b). Previous studies have shown that exogenous administered protein oligomers and aggregates are taken up by cultured cells (including cultured neurons) and then induce oligomerization and/or aggregation of protein produced endogenously in the cultured cells. This process is termed “seeding” and has been demonstrated to occur with a wide range of aggregates including tau (oligomers and fibrils), α-synuclein (oligomers and fibrils), SOD1 and prion protein [1, 30, 40–42].

**Fig. 1:**
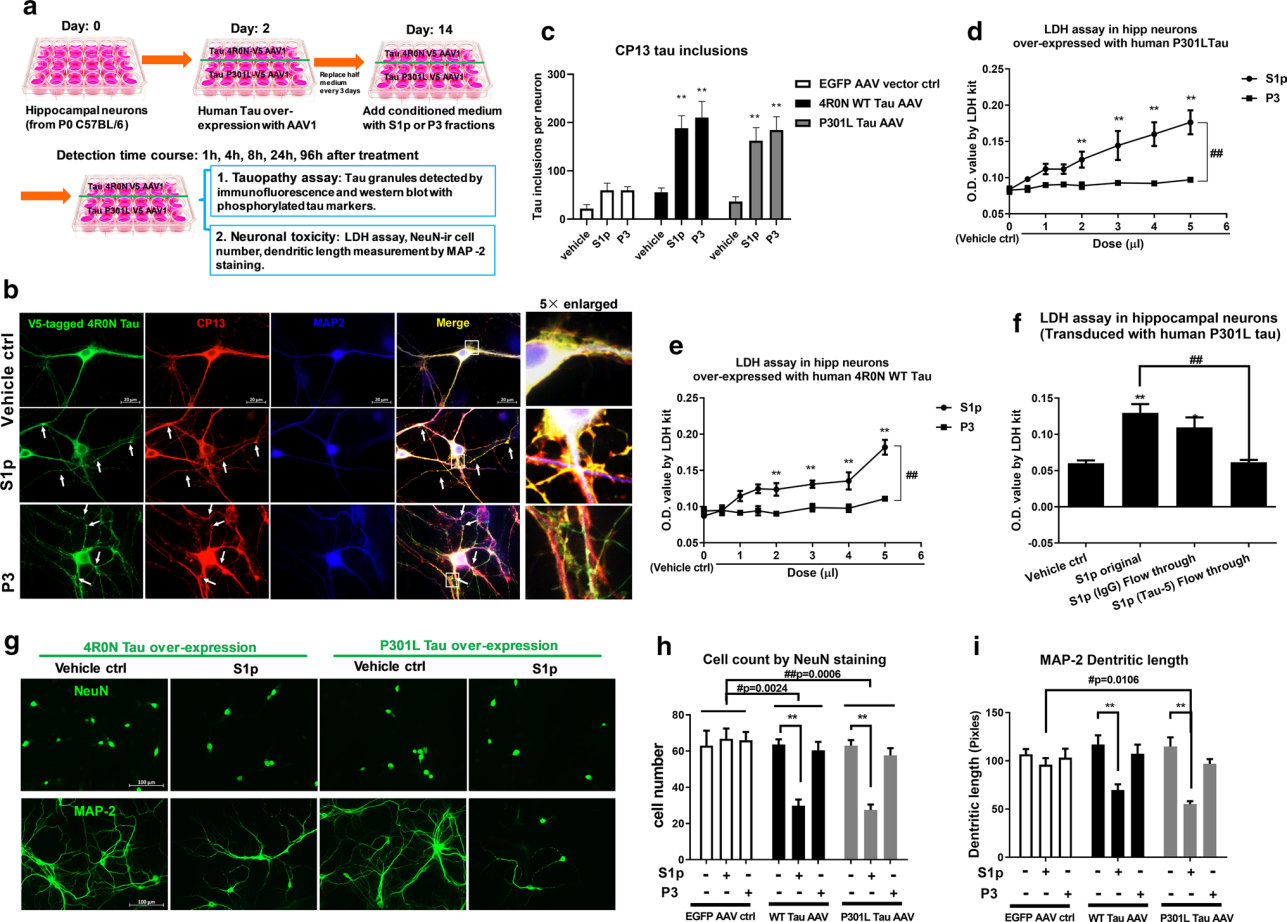
Oligomeric tau fractions trigger neurotoxicity. **a** Diagram of experiment design using C57BL/6 primary hippocampal cultures, including plating, transduction, treatment and harvest in time course. **b** Representative images showed the co-localization (yellow) of CP13 (red) marked phosphorylated tau with V-5 (green) tagged tau at 24 h after S1p or P3 fraction treatment. Scale bar 20 µm. **c** quantitative analysis of tau aggregation induced by the S1P and P3 fractions. The number of tau inclusions was quantified by Image J. Data expressed as average number of tau inclusions per neuron. **p < 0.001 compared to their corresponding vehicle control in EGFP-AAV vector, WT tau and P301L tau over-expression groups. **d**–**e** The LDH assay showed dose-dependent toxicity in hippocampal neurons over-expressed human 4R0 N tau or P301L upon S1p treatment. Toxicity was not elicited by treatment with P3 fractions, ##*p *< 0.001 in the linear comparison of S1p and P3, **p < 0.001 compared to the corresponsive vehicle control. **f** The LDH assay of neurons treated with vehicle or S1p fractions ± immuno-depletion of tau, **p = 0.0011, *p = 0.0148 compared to vehicle control, ^##^*p *= 0.0014 compared to original S1p treated neurons. **g** Representative images showing the cell loss (NeuN) and shorter dendritic length (MAP-2) at 96 h after S1p treatment. Scale bar 100 µm. **h** Quantification of NeuN-positive cell number by image J. Cell number equals total count per 5 images each well. **p < 0.001 compared to corresponsive vehicle control. WT tau over-expression VS EGFP vector control, #*p *= 0.0024; P301L tau over-expression VS EGFP vector control, ##*p *= 0.0006. **i** Measurement of dendritic length at 96 h after S1p or P3 treatment. **p < 0.001 compared to corresponsive vehicle control. In S1p treated groups, cells of P301L tau over-expression VS EGFP vector control, #*p *= 0.0106. Data are represented as mean ± SEM. Independent experiments were repeated at least 4 times with triplicate wells each time

Primary cultures of hippocampal neurons from C57BL/6 mice were transduced with human 0N4R WT or P301L tau by V5-tagged AAV1 at 2 days in vitro (DIV), as diagrammed in Fig. 1a. On DIV 14, neurons were treated with conditioned medium containing different doses of S1p or P3 fractions. After 24 h of treatment, the neurons were fixed and immunolabeled using an anti-V5 antibody to detect tau produced in the neurons, and CP13 (detecting pS202) to detect hyperphosphorylated tau. Both S1p and P3 fractions triggered robust responses of the cell synthesized tau (Fig. 1b–c). Moreover, in comparison of AAV1 expressing tau to a AAV1 vector only expressing EGFP in primary neurons, we found that the propagated tau inclusions induced by S1p or P3 were much related to the level of tau synthesized in the recipient neurons (Fig. 1c and supplemental Fig. 2), which is also consistent with previous study in tau knockout neurons [49].

To compare toxicities of the S1p and P3 fractions, we treated the cultures as above and after 24 h collected the culture media for the analysis of lactate dehydrogenase (LDH) release. The LDH data showed that S1p induced dose-dependent toxicity that was statistically significant beginning at the 2-µl dose, whereas P3 did not trigger cell injury in cultures transduced with either WT or P301L tau, even at the highest doses (Fig. 1d–e).

We proceeded to test whether the toxic species in the oligomeric tau S1p fraction was, in fact, tau. First, we incubated the S1p fraction with tau-5 (recognizing total tau) or IgG control antibody resins to pre-adsorb tau, and then we tested the toxicity of the S1p eluate on the toxicity of hippocampal neurons grown in cell culture. Primary hippocampal neurons over-expressing human P301L Tau (by AAV1 mediated transduction) were treated for 24 h with oligomeric S1p fractions or tau-depleted S1p fractions. The supernatant was then collected for the measurement of LDH. We observed a striking reduction of LDH release from cultures exposed to S1p fractions absorbed with Tau5 compared to the IgG (Fig. 1f). These results indicate that the toxicity of the S1p fraction results primarily from tau, which is predominantly oligomeric in this fraction. The efficiency of tau-depletion from S1p fractions was confirmed by western blot as shown in supplemental Fig. 1c.

Neuronal toxicity was further quantified with NeuN and MAP-2 immunofluorescence labeling of treated neurons at 96 h after addition of the S1p or P3 fractions. Quantification of NeuN-positive cells showed that the S1p fraction (2 μl) induced more than 50% neuron loss (saline 60.6 ± 4.3 vs S1p 29.8 ± 3.4 in WT tau neurons, *p *< 0.001 and saline 61.0 ± 4.8 vs S1p 27.4 ± 4.1 in P301L tau neurons, *p *< 0.001) (Fig. 1g–h). In contrast, the P3 fraction (2 μl) did not elicit any neuron loss. Measurement of dendritic length showed similar results. Quantification of dendritic length with MAP-2 labeling revealed that neurons treated with the S1p fraction had a ~ 40% reduction of dendritic length compared to vehicle control, while neurons treated with the P3 fraction exhibited no reduction in neurite length (saline 119.7 ± 16.4 vs S1p 64.0 ± 5.9 in WT tau neurons, *p *< 0.001 and saline 116.3 ± 14.9 vs S1p 55.0 ± 4.9 in P301L tau neurons, *p *< 0.001) (Fig. 1g, i). These results demonstrate that oligomeric tau is toxic, while fibrillar tau exhibits no detectable toxicity.

### Propagated oligomeric and fibrillar tau species accumulate in separate neuronal compartments in vivo

**Fig. 2:**
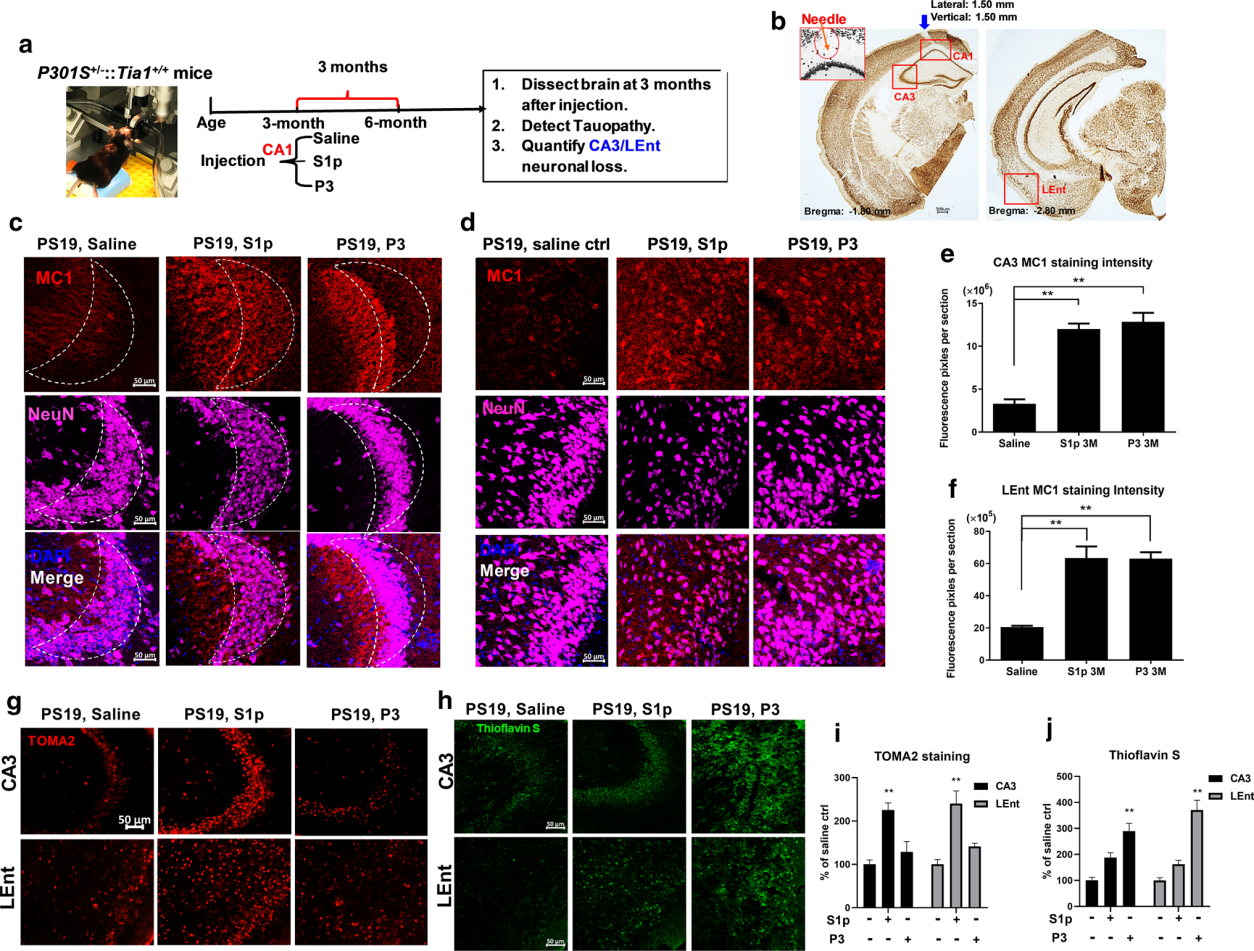
Propagated oligomeric and fibrillar tau species accumulate in separate neuronal compartments in vivo. **a** The design for the *in vivo* tau propagation experiments. The saline ctrl, S1p or P3 fractions were stereotaxically injected into the CA1 region of PS19 mice at 3 months of age. The mice were then aged three more months, and then killed at 6 months, whereupon the brains were harvested to assess tauopathy and neuronal loss. **b** IHC of DAB stained brain sections showing the position of injection site (inset coordinates: 1.80 mm posterior and 1.50 mm lateral to bregma; 1.50 mm ventral to cortical surface). The red boxes highlight the CA1 injection site, as well as the sites used for analysis, which include the neighboring CA3 site and the lateral entorhinal cortex (LEnt, bregma − 2.80 mm), a remote projection site. Shown are coronal sections at two z-coordinates (corresponding to neurons that were labelled for tauopathy and neurotoxicity). Scale bar 300 µm. **c** Images showing the S1p- and P3-induced hyperphosphorylated tau inclusions in CA3: MC1 (red) staining, neuronal marker, NeuN (violet), and DAPI (blue). Scale bar 50 µm. **d** Images showing the S1p- and P3-induced hyperphosphorylated tau inclusions in LEnt with detection of MC1 (red), NeuN (violet) and DAPI (blue). Scale bar 50 µm. **e** Quantification of the MC1 fluorescence intensity in the CA3. *N* = 4 (average of two brain slice from each mouse), saline vs S1p, **p < 0.001; saline vs P3, **p < 0.001. No difference observed between S1p and P3, *p *= 0.8467. **f** Quantification of the MC1 fluorescence intensity in the LEnt. *N* = 4 (average of two brain slice from each mouse), saline vs S1p, **p < 0.001; saline vs P3, **p < 0.001. No difference between S1p and P3, *p *= 0.9979. **g** Representative images of TOMA2 (labeling oligomeric tau) staining detect tau oligomers’ accumulation in CA3 and LEnt after S1p or P3 injection. **h** Representative images of thioflavin S staining in CA3 and LEnt detect tau fibrils accumulation after S1p or P3 injection. **i** Quantitative analysis of TOMA2 staining in CA3 and LEnt after S1p or P3 injection, respectively. **p < 0.001 compared to saline control. **j** Quantitative analysis of thioflavin S staining in CA3 and LEnt after S1p or P3 injection, respectively. **p < 0.001 compared to saline control. Data are represented as mean ± SEM

Having documented the effects of the tau fractions on propagation and toxicity in primary neuron cultures, we proceeded to examine the effects in vivo. To compare the propagation ability of oligomeric and fibrillary tau species and investigate their co-relation to neurotoxicity, we stereotaxically injected S1p or P3 fractions into the CA1 region of the hippocampus of 3-month-old PS19 mice. Three months later, tau pathology was examined in neighboring hippocampal CA3 neurons, and in the distant, but anatomically linked, projection target neurons of lateral entorhinal cortex (LEnt) (Fig. 2a–b) [7]. Our result showed that both S1p and P3 significantly elevated tau aggregation in neighboring CA3 neurons and network LEnt neurons, as detected by the conformational tau marker MC1 (epitope within aa 312–322) (Fig. 2c–f). However, the phenotypes elicited by the S1p and P3 fractions were different; the S1p mostly increased tau accumulation in neuronal soma while the P3 induced tangles in dendrites and synapses (Fig. 2c–d). To further clarify the cell type specificity of tau accumulation in S1p- or P3-injected mice, we performed the co-staining of CP13 (hyperphosphorylated tau, site pS202), MAP-2 (dendritic marker for neuron), GFAP (marker for astrocyte) or Iba-1 (marker for microglia) in the mice brain sections 3 months after S1p or P3 injection. Our results indicate that the tau propagation induced by the S1p or P3 fractions occurred among neurons and rarely in glial or microglial cells (supplemental Fig. 4).

To further confirm the existence of oligomeric and fibrillar forms of tau in S1p or P3 injected mice brain, tau oligomer marker TOMA2 and fibrillar tau marker thioflavin S were used to detect tau inclusions in CA3 and LEnt, respectively. The data indicate that the S1p fraction primarily induced oligomeric tau aggregation, while the P3 fraction primarily induced fibrillar tau aggregation (Fig. 2g–j).

To exclude the possibility that tau fractions diffuse in the brain instead of propagating from the injection site located in the CA1 hippocampal neurons to the neighboring CA3 region of the hippocampal, and the remote projections in the LEnt, we conjugated the S1p fractions with Alexa Fluor-488 fluorophores and studied the distribution of the conjugated, injected tau in a separate cohort of WT mice and PS19 mice. The injected C57BL/6 wild-type mice were killed at 3 months after injection (litter mate of and same time line as to PS19 mice). We also examined a cohort of injected PS19 mice that were killed at 15 days after injection. In both cohorts, the fractions containing tau aggregates exhibited only modest diffusion into CA3 and no diffusion into distant LEnt region (supplemental Fig. 3). The limited diffusion of the tagged tau suggests that the tau aggregation present in CA3 and LEnt over both short- and long-term models resulted predominantly from the templating of human tau synthesized in the recipient neurons.

### Oligomeric Tau co-localized with TIA1 positive granules in soma of neurons

**Fig. 3:**
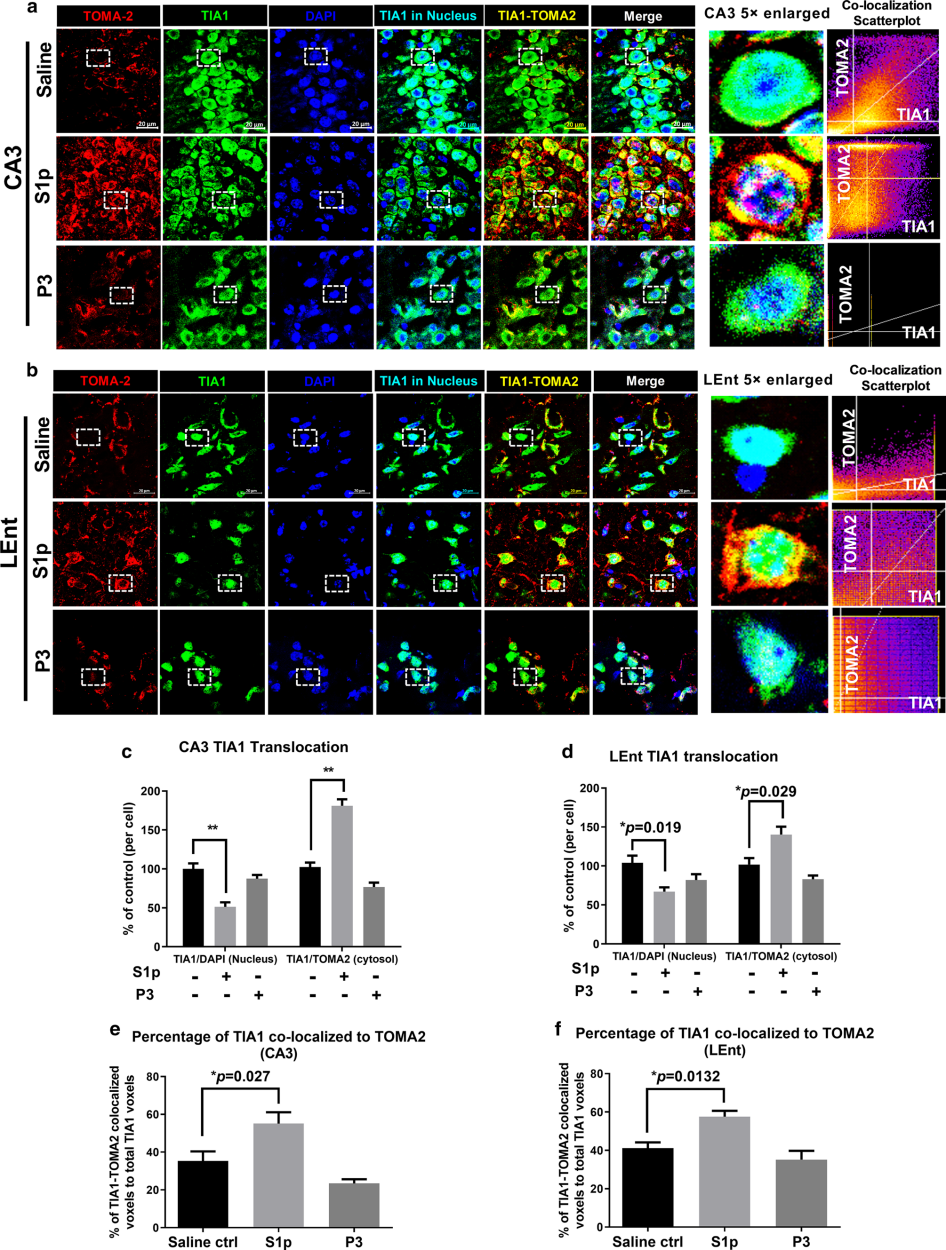
Oligomeric Tau co-localized with TIA1 positive granules in soma of neurons. **a**–**b** Representative images of CA3 (a) and LEnt **b** for oligomeric tau marker TOMA2 (red) staining overlapped with TIA1 (green) and DAPI (blue) at 3 months after injection. Scale bar 20 µm. The nucleus TIA1 was masked by DAPI (light blue) and the cytosolic TIA1 co-localized with oligomer tau was masked by TOMA2 (yellow). The 5 × enlarged single cell images showed the localization of TIA1 in nucleus (light blue) or cytosol (green or yellow). And the co-localization intensity of TIA1 to TOMA2 was highlighted by scatterplot. **c**–**d** Translocation of TIA1 from nucleus to cytosol in neurons of CA3 **c** and LEnt **d** was quantified by DAPI masked TIA1 in nucleus and TOMA2 masked TIA1 in cytosol. The number of colocalized voxels of TIA1 to DAPI or TIA1 to TOMA2 in each neuron of saline group was normalized into 100%, and comparison made between S1p- or P3-treated animals to Saline. In CA3, **p < 0.001 S1p compared to saline. In LEnt, *p = 0.019 and *p = 0.029 comparing S1p-treated animals to saline group of TIA1-DAPI co-localization and TIA1-TOMA2 co-localization, respectively. **e**–**f** Quantification for the percentage of TIA1 that co-localized with TOMA2 to its total voxels, in CA3 neurons **e** and LEnt neurons **f** respectively. S1p compared to saline, *p = 0.027 in CA3, *p = 0.0132 in LEnt. Analysis of TIA1-DAPI or TIA1-TOMA2 co-localization was by Image J Fiji coloc 2 plugin. *N* = 15 (15 neurons from 3 sections of 3 mice). Data are represented as mean ± SEM

The mechanism of propagation and toxicity in neurons is largely unknown. The localization of propagated tau oligomers to neuronal soma raised the possibility that they might interact with the TIA1/SG pathway, which also localizes to the neuronal soma. To test for co-localization of tau oligomers with TIA1 positive SGs, brain tissue sections from mice treated with the S1p or P3 fractions were probed with anti-TIA1 antibody and the oligomeric tau-specific antibody TOMA2 (Fig. 3a–b). Under basal conditions, TIA1 is nuclear and co-localized with the DAPI (identifying nuclear DNA), while under stressed conditions TIA1 translocates to the cytoplasm where it did not co-localize with DAPI. First, we determined the effects of the S1p treatment on nuclear localization of TIA1 by creating a nuclear mask corresponding to DAPI reactivity (Fig. 3a–c); we observed that brains treated with S1p exhibited a striking reduction in fraction of neurons exhibiting co-localization of TIA1 with DAPI, suggesting that the propagated S1p induced translocation of TIA1 to the cytoplasm (Fig. 3c, d; TIA1/DAPI bars). Next, we determined the fraction of neurons exhibiting co-localization of TOMA2 with TIA1 in somatic regions outside the DAPI imaging mask (Fig. 3a–d, TIA1/TOMA2 bars); we observed that brains treated with S1p exhibited a striking increase in cytoplasmic TIA1/TOMA2 overlap (Fig. 3a–d). Note that the TIA1/TOMA2 values in Fig. 3e, f correspond to the size of the area in the lower left quadrant of the scatterplots in Fig. 3a, b. We also quantified these data by calculating the number of voxels corresponding to cytoplasmic TIA1, the number of voxels where TIA1 and TOMA2 showed overlap, and then determining the ratio of overlapped TOMA2/TIA1 voxels to cytoplasmic TIA1 voxels in CA3 and the LEnt (Fig. 3e, f). The strong co-localization between propagated oligomeric tau and TIA1 contrasted strongly with the results for propagated fibrillar tau, which did not co-localize significantly with TIA1 (Fig. 3a–f).

The strong co-localization between oligomeric tau and TIA1 might result from the ability of oligomeric tau to induce cytoplasmic translocation of TIA1 either due to stress and/or a direct ability of oligomeric tau to bind TIA1 [2]. The close physical connection between oligomeric tau and TIA1 is highlighted by the 3D image generated from Z-stacks showing co-localization of TIA1-TOMA2 in the cytosol (supplemental Fig. 5). These results suggest that TIA1 contributes to the pathophysiology of propagated oligomeric tau. Based on these observations, we hypothesized that TIA1 might also contribute to the neurodegeneration associated with the propagation of oligomeric tau.

### The propagated oligomeric tau fraction induces neuron loss

**Fig. 4:**
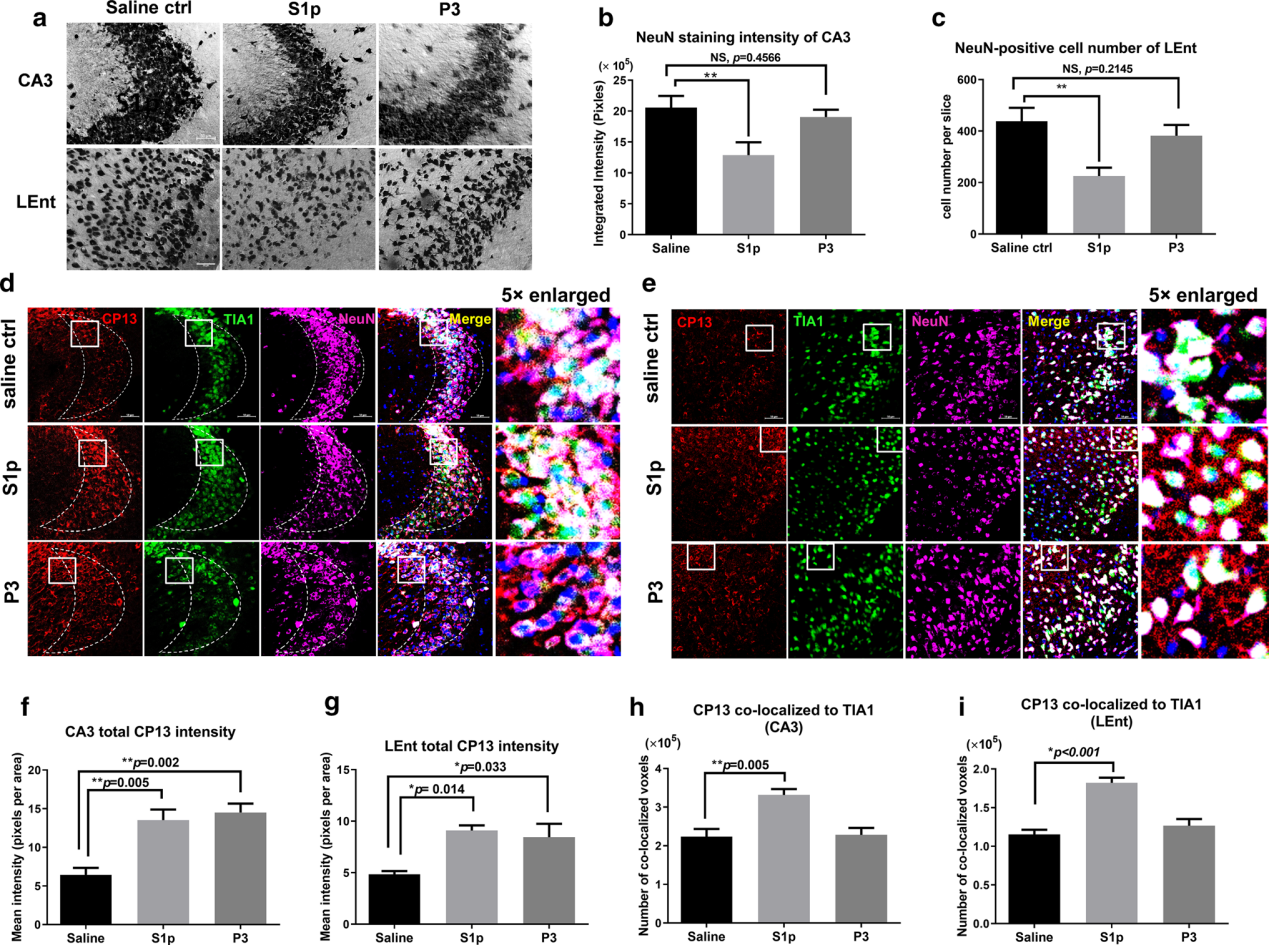
The propagated oligomeric tau fraction induces neuron loss. **a** Images show the immunohistochemistry DAB labeling of NeuN-positive neurons in CA3 and LEnt at 3 months after injection. Scale bar 50 µm. **b** Quantification of NeuN staining intensity of CA3 by Image J. *N* = 4 (average pixels of two sections from each mouse), **p = 0.0004. No difference observed between P3 and saline, *p *= 0.4566. **c** Cell count of NeuN-positive neurons in LEnt by Image J automatic count. *N* = 4 (average number of two sections from each mouse), **p = 0.0002. No difference observed between P3 and saline, *p *= 0.2145. **d**-–**e** Images represent the CP13 (red) marked phosphorylated tau co-localization (yellow) with RBP TIA1 (green), neuronal marker NeuN (violet) and DAPI (blue) of CA3 **d** and LEnt **e** after 3 months of injection, respectively. Scale bar 50 µm. **f**–**g** Quantification of CP13 staining intensity in CA3 **f** and LEnt **g**. Data showed by the mean pixels per area of each quantified section, *N* = 4 (average of two sections from each animal). In CA3 **f**, **p = 0.005 S1p compared to saline and **p = 0.002 P3 compared to saline. In LEnt **g**, *p = 0.014 S1p compared to saline and *p = 0.033 P3 compared to saline. **h**–**i** Quantification for the number of co-localized voxels in CP13-TIA1 overlap (as shown yellow in **d**, **e**). Data showed by the total number of co-localized voxels in each quantified brain section, *N* = 4 (average of two sections from each animal). In CA3 **h**, **p = 0.005 S1p compared to saline. In LEnt **i**, *p < 0.001 S1p compared to saline. Multiple comparison test by Tukey’s. Data are represented as mean ± SEM

As described above, the S1p fraction elicited neurotoxicity in cultured primary neurons, whereas the P3 fraction did not. We proceeded to test whether the neurodegeneration in vivo induced by the propagated tau was greater for oligomeric (S1p fraction) tau than fibrillar tau (P3 fraction). Neurodegeneration associated with propagation of the oligomeric or fibrillar tau was assessed by immunohistochemical labeling with neuronal marker NeuN in CA3 and LEnt regions (Fig. 4a). The result showed that S1p induced more than 40% neuronal loss in CA3 and more than 55% reduction NeuN-positive cell number in LEnt (Fig. 4b–c). As predicted from the in vitro experiments, the P3 fraction did not induce significant neuronal loss in CA3 or Lent within 3 months, despite inducing extensive accumulation of hyperphosphorylated tau (CP13 staining) in the dendrites (Fig. 4d–g). These data suggest that the S1p fraction (containing predominantly tau oligomers) is less toxic than the P3 fraction (containing predominantly tau fibrils); however, it is possible that the P3 fraction might exhibit some toxicity over a longer time course.

We hypothesized that the neurodegeneration induced by the propagated oligomeric tau might elicit a stress reaction in the affected neurons and that this stress response might be associated with cytoplasmic translocation of TIA1 and SG formation. This is important because recent studies indicate that tau pathology associates with TIA1 positive SG responses in neurons [2, 43, 44]. We proceeded to examine whether the tau pathology elicited by propagation of oligomeric tau co-localized with TIA1 and other SG markers. Note that in these experiments, we took advantage of the ability of phospho-tau antibodies to detect the tau pathology propagated by the S1p fraction; TOMA2 only detects oligomeric tau, while phospho-tau antibodies detect both oligomeric and fibrillar tau. Tissue sections were labeled with markers of phospho-tau (CP13, PHF1) and SGs (TIA1 and eIF3ŋ). Robust co-localization of cytoplasmic tau/TIA1 granules was observed in CA3 and LEnt by CP13 and TIA1 co-staining (Fig. 4d–e, h–i). The additional phospho-tau marker PHF1 also indicated a strong co-localization of tau granules to TIA1 in S1p-induced tau propagation as shown in supplemental Fig. 6a. Demonstration that these granules were genuine SGs was supported by co-localization of the S1p-propagated tau with the SG marker eIF3ŋ (supplemental Fig. 6b). These results indicate that tau propagated by oligomeric tau fractions co-localized with TIA1 positive SGs in the neuronal soma of the CA3 and LEnt regions of the brain.

### TIA1 reduction prevents tau propagation and toxicity from the oligomeric tau fraction

**Fig. 5:**
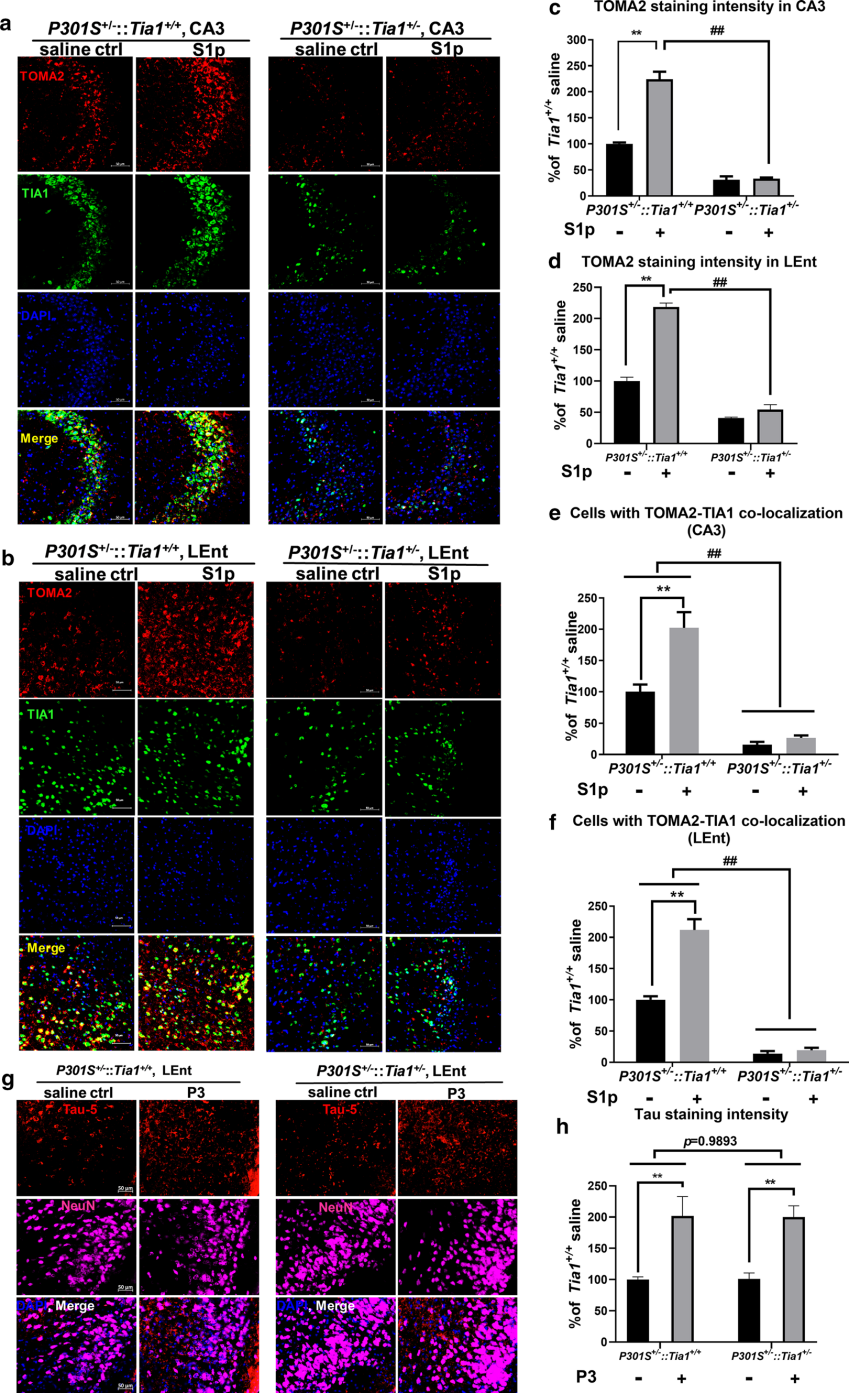
TIA1 reduction prevents tau propagation from the oligomeric tau fraction. **a**–**b** Representative images showing co-localization of TOMA2 (red) and TIA1 (green) in CA3 **a** and LEnt **b** of PS19 *Tia1*^+/+^ and PS19 *Tia1*^+/−^ mouse brain, respectively, 3 months after injection in CA1of saline or S1p. With the co-staining, total cell nuclei are stained with DAPI (blue). Scale bar 50 µm. **c**–**d** Quantitative analysis of TOMA2 staining intensity of CA3 and LEnt in PS19::*Tia1*^+/+^ and PS19::*Tia1*^+/−^ mice, respectively. In PS19::*Tia1*^+/+^ mice, S1p VS saline, **p < 0.001; With S1p injection, PS19::*Tia1*^+/−^ VS PS19::*Tia1*^+/+^, ##*p *< 0.001. **e**–**f** Quantification of cells with TOMA2-TIA1 co-localization. The number of cells with co-localization in the saline group of *Tia1* wild-type animals was normalized to 100 for CA3 **e** and LEnt **f**, respectively. For CA3 **e**, in PS19::*Tia1*^+/+^ mice, S1p compared to saline, **p < 0.0001; in PS19::*Tia1*^+/−^ mice, S1p compared to saline, no difference observed, *p *= 0.6019. Genotype difference ##*p *< 0.0001. For LEnt **f**, in PS19::*Tia1*^+/+^ mice, S1p compared to saline, **p < 0.0001; in PS19::*Tia1*^+/−^ mice, S1p compared to saline, no difference was observed, *p *= 0.6922. Genotype difference ##*p *< 0.0001. **g**–**h** Representative images of tau-5 staining (red) showed that fibrillary tau in P3 fractions propagated into LEnt and elevated tau inclusions equally in *P301S*^+*/*−^:: *Tia1*^+*/*+^ and PS19::*Tia1*^+/−^ mice. **p < 0.001 compared to saline control within the corresponding genotype. Gene factor p = 0.9893 between *P301S*^+*/*−^:: *Tia1*^+*/*+^ and PS19::*Tia1*^+/−^ mice. Scale bar 50 µm. Data are represented as mean ± SEM, *N* = 4 (average of two sections from each animal)

The association of TIA1 with propagated oligomeric tau suggests the possibility that TIA1 reduction might protect against neurodegeneration from tau propagation in a manner similar to that observed in our prior studies [2, 43]. To test the role of TIA1 in the propagation and toxicity of oligomeric tau, S1p fractions containing oligomeric tau aggregates were injected into 3-month-old PS19::*Tia1*^+*/*+^ and PS19::*Tia1*^+*/*−^ mice and harvested at 6 months. The TOMA2 staining data showed that TIA1 reduction greatly reduced the basal level of TOMA2 oligomeric tau in PS19::TIA^+/−^ mice, as well as blocking the propagation of oligomeric tau in S1p-injected mice (Fig. 5a–d). The number of neurons with TOMA2-TIA1 co-localization was significantly reduced in CA3 and LEnt of PS19::*Tia1*^+*/*−^ mice compared to PS19::*Tia1*^+*/*+^ mice (Fig. 5e–f). However, in contrast to the effect of TIA1 reduction on S1p propagation, fibrillary tau in P3 fraction still increased similar levels of tau inclusions in LEnt of *P301S*^+*/*−^:: *Tia1*^+*/*−^ mice compared to *P301S*^+*/*−^:: *Tia1*^+*/*+^. These results suggest that there is no effect of TIA1 reduction on fibrillary tau propagation and that there is a specific action of TIA1 on regulating the propagation of oligomeric tau (Fig. 5g–h).

**Fig. 6:**
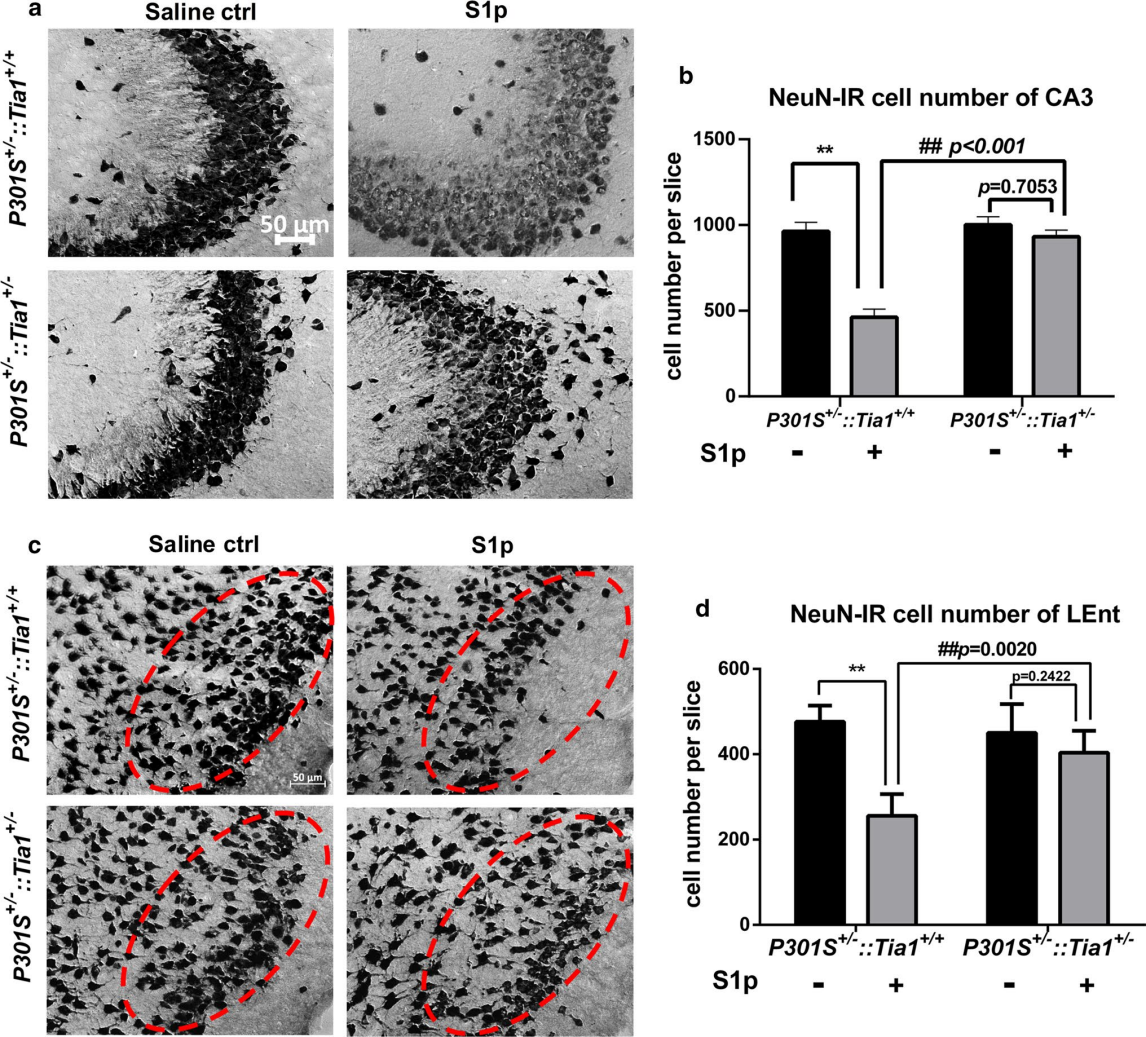
TIA1 reduction prevents neurotoxicity induced by oligomeric tau propagation. **a** Representative NeuN-positive images showed the DAB immunohistochemical staining of CA3 at 3 months after injection of saline or S1p in PS19 TIA1 heterozygous mice compared to PS19 TIA1 wild type. Scale bar 50 µm. **b** Count of NeuN-positive cell number from DAB stained CA3 sections in **a**, **p < 0.001 compared to corresponsive saline control in PS19::*Tia1*^+/+^. In PS19::*Tia1*^+/−^ mice, S1p compared to saline, *p *= 0.7053. Under S1p injection, compare PS19::*Tia1*^+/−^ to PS19::*Tia1*^+/+^ mice, ##*p *< 0.001. **c** Representative NeuN-positive images showed the DAB immunohistochemical staining of LEnt at 3 months after injection of saline or S1p in PS19 TIA1 heterozygous mice compared to PS19 TIA1 wild type. The red ovals identify similar areas in each tissue section and highlight loss of neurons in the PS19 TIA1 wild-type mouse, with rescue in the PS19 TIA1 heterozygous mouse. Scale bar 50 µm. **d** Count of NeuN-positive cell number from DAB stained Lent sections in **c**, **p < 0.0001 compared to corresponsive saline control in PS19::*Tia1*^+/+^. In PS19::*Tia1*^+/−^ mice, S1p compared to saline, *p *= 0.2422. Under S1p injection, compare PS19::*Tia1*^+/−^ to PS19::*Tia1*^+/+^ mice, ##*p *= 0.0020. Data are represented as mean ± SEM. *N* = 4; average cell number of three sections from each mouse was used. Data analysis was by two-way ANOVA, post hoc multiple comparison test by Fisher’s LSD

To further investigate whether oligomer mitigation by TIA1 reduction could retrieve S1p-induced neuronal loss, we measured the neuronal number of CA3 and LEnt in S1p injected *P301S*^+*/*−^:: *Tia1*^+*/*−^ and *P301S*^+*/*−^:: *Tia1*^+*/*+^ mice. Our data showed that PS19::*Tia1*^+*/*−^ mice exhibited significantly higher numbers of NeuN-positive neurons in CA3 and LEnt, respectively, compared to *P301S*^+*/*−^:: *Tia1*^+*/*+^ mice at 3 months after S1p injection (Fig. 6a–d). These results indicated that TIA1 reduction exerts neuroprotection against tau propagation, following exposure to extracellular oligomeric tau.

### Silencing of TIA1 expression protects against oligomeric Tau toxicity

**Fig. 7:**
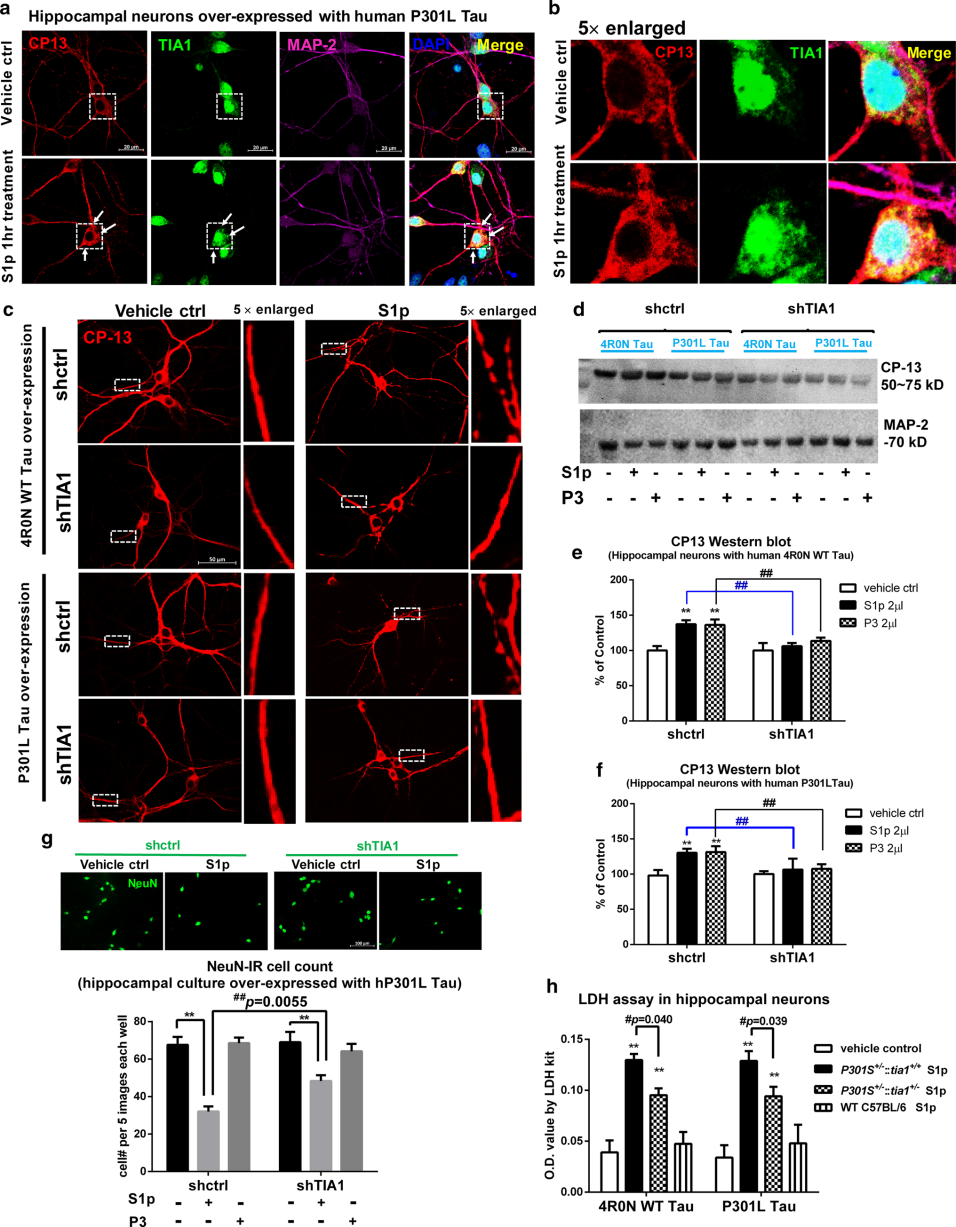
Silencing of TIA1 expression protects against oligomeric Tau toxicity. **a** Representative images showing the co-localization of phosphorylated tau inclusions CP13 (red) with TIA1 granules (green) in hippocampal neurons over-expressing human P301L tau, at 1 h after S1p or vehicle treatment. Co-labeled markers are MAP-2 (violet) for neurons and DAPI (blue) for nuclear. Scale bar 20 µm. **b** To show the translocation of TIA1 from nuclear to cytosol and co-localization with tau inclusions in neuronal soma, high-magnification images were enlarged fivefold from the boxed neurons in **a**. **c** CP13 staining representative images show that TIA1 knock down disperses S1p induced bead-like tau granules in synapse and dendrites, creating a more continuous, less consolidated distribution of CP13 positive tau. CP13 staining of hippocampal cultures over-expressing human 4R0 N WT or P301L tau fixed at 24 h after treatment. **d** TIA1 knockdown prevents the elevation of CP13 phospho-tau from S1p or P3 treatment. Immunoblots of primary neuron lysates expressing either 4R0N WT or P301L tau, knocked down with either shCtrl or shTIA1 and treated for 24 h with S1p or P3 fractions. Phosphorylated tau detected by CP13 and MAP-2 internal control. **e** Quantification of CP13 immunoblot in cell lysate of hippocampal cultures over-expressing human 4R0 N WT tau, transduced with shTIA1 or shCtrl AAV on day- 4, treated with vehicle control, S1p or P3 on day 14 and harvested at 24 h after treatment. *N* = 3, in shCtrl, S1p compared to vehicle **p < 0.001, P3 to vehicle **p < 0.001. For S1p treatment, shTIA1 compared to shCtrl, ##*p *= 0.001. For P3 treatment, shTIA1 compared to shCtrl, ##*p *= 0.0016. **f** Quantification of CP13 immunoblot in cell lysate of hippocampal cultures over-expressing human P301L tau, transduced with shTIA1 or shCtrl AAV on day 4, treated with vehicle control, S1p or P3 on day 14 and harvested at 24 h after treatment. *N* = 3, in shctrl, S1p compared to vehicle **p = 0.007, P3 to vehicle **p = 0.006. For S1p treatment, shTIA1 compared to shCtrl, ##*p *= 0.006. For P3 treatment, shTIA1 compared to shCtrl, ##*p *= 0.006. Two-way ANOVA multiple comparison test by Fisher’s LSD. **g** NeuN-positive neurons in shctrl and shTIA1 cultures after saline or S1p treatment. **p < 0.0001 compared to corresponding control, ##*p *= 0.0055 compared to S1p group in shctrl cultures. **h** LDH assay with conditioned medium at 24 h after vehicle or S1p treatment in hippocampal neurons over-expressed with human WT or P301L Tau. The S1p fractions are from three genotypes of 9-month-old brains, including PS19 *Tia1*^+/+^, PS19 *Tia1*^+/−^ and C57BL/6 wild-type mice. *N* = 4 separate times of experiment (triplicate wells each time). **p < 0.001 compared to corresponsive saline control. In WT tau over-expressed neurons, *Tia1*^+/+^ S1p compared to *Tia1*^+/−^ S1p, #*p *= 0.0402. In P301L tau over-expressed neurons, *Tia1*^+/+^ S1p compared to *Tia1*^+/−^ S1p, #*p *= 0.0394. Two-way ANOVA, post hoc multiple comparison test by Fisher’s LSD. Data are represented as mean ± SEM

Next, we used primary neuron cultures to test whether the depletion of TIA1 from neurons protects against oligomeric tau fractions in a cell-autonomous manner. We also tested whether TIA1 reduction in the target neurons protects against oligomeric tau fractions from PS19::*Tia1*^+*/*+^ mice. First, we transduced cultures of hippocampal neurons with V5-tagged tau virus and exposed the DIV-14 cultures to S1p fractions from PS19::*Tia1*^+*/*+^ mice, as described in the experiments presented in Fig. 1. We confirmed that the S1p seeding induced tau inclusions in soma that were positive for CP13 and demonstrated that these inclusions co-localized with TIA1 in cell body of P301L tau over-expressed neurons (Fig. 7a–b). Next, TIA1 expression was silenced by expressing shRNA against *Tia1* (shTIA1) via AAV9-mediated transduction in hippocampal neurons over-expressing human WT or P301L tau. The efficiency of TIA1 knock down was greater than 50% of TIA1 as detected in cell lysate by immunoblot (supplemental Fig. 7a–b). We documented propagation of S1p-induced tauopathy (CP13 staining) in these cultures (Fig. 7c, supplemental Fig. 7c–d) and then used immunoblotting to quantify seeded tau using the pTau antibody CP13; these studies demonstrated that TIA1 knock down reduced levels of pTau induced by S1p seeding (Fig. 7d–f, supplemental Fig. 7c–d). We also examined the effects of TIA1 knockdown on neuronal survival using NeuN staining. Analysis of neuronal survival by NeuN demonstrated that TIA1 knock down reduced neuronal death by 30% (Fig. 7g). These results indicate that reduction of endogenous TIA1 provides neuroprotection against propagated tau.

We also performed the converse experiment of testing the effects of S1p fractions from PS19::*Tia1*^+*/*−^ mice. Our previous results showed that TIA1 reduction decreases the amount of tau oligomer (which localizes to the S1p fraction). We proceeded to test whether the S1p fraction from PS19::*Tia1*^+*/*−^ mice was less toxic than the equivalent fraction from PS19::*Tia1*^+*/*+^ mice. We exposed DIV14 cultures of hippocampal neurons to samples of S1p fraction isolated from PS19::*Tia1*^+*/*+^, PS19::*Tia1*^+*/*−^ and WT C57BL/6 mice. Medium was collected after 24 h. Analysis of LDH levels from the medium of the neurons demonstrated a 30% decrease in LDH from cultures exposed to PS19::*Tia1*^+*/*−^ S1p compared to PS19::*Tia1*^+*/*+^ (Fig. 7h). These results provide support for the hypothesis that protection by TIA1 depletion results from the reduction of oligomerization of tau in both the donor of the S1p seeding tau and in the recipient neurons in which endogenous tau pathology is seeded.

## Discussion

Seeding and propagation of pathological proteins is a phenomenon that has been robustly observed, but much of the additional biology is unknown. Seeding has been shown for many types of pathology including tau, β-amyloid, α-synuclein, TDP-43 and SOD-1 [11, 13, 14, 17, 25, 33, 35, 45, 46]. The mechanisms of propagation have been studied extensively and include proposed mechanisms such as direct secretion, exosomes and tunneling nanotubes [12]. Most studies of tau propagation, though, focus on the presence and distribution of tau pathology rather than on the mechanism of neurodegeneration mediated by propagated tau [16]. The current manuscript rigorously assesses the relationship between tau aggregate structure, propagation and neurodegeneration. This manuscript reports the pivotal discoveries that propagated oligomeric tau forms’ inclusions in the soma that co-localized with TIA1 and other SG markers and that reducing TIA1 decreased neurodegeneration induced by propagated oligomeric tau. These results provide fundamental advances to our understanding of the mechanisms of tau propagation and demonstrate that reducing TIA1, a core nucleating SG protein, protects against the deleterious effects of tau propagation.

Consistent with previous studies, we observed that the structure of the propagated tau aggregates determined the resulting pattern of tau pathology [31, 36]. Oligomeric tau accumulated in neuronal soma in the CA3 and LEnt regions, while fibrillar tau showed a distinct accumulation in the dendritic processes. This pattern was evident in the CA3 hippocampal region, which is closer to the CA1 injection site, as well as in the LEnt, which is distant from the injection site. The distance of the tau pathology in the CA3 and LEnt regions from the CA1 injection site meant that much of the oligomeric and fibrillar tau accumulating in the CA3 and LEnt regions resulted from templating of propagated tau; the absence of the injected tau was proven by labeling aliquots of injected tau with fluorophores and demonstrating the absence of fluorescently labeled tau in the CA3 and LEnt regions.

Interestingly, the S1p fraction obtained from PS19::*Tia1*^+*/*−^ mice exhibited very little toxicity, very little propagated tau and little co-localization of propagated tau with SG markers. The paucity of toxicity in this fraction reflects a paucity of tau oligomers. In contrast, the S1p fraction from PS19::*Tia1*^++^ mice has abundant tau oligomers which are highly toxic in vitro and in vivo. We had previously observed that PS19::*Tia1*^+*/*−^ mice exhibit lower levels of tau oligomers; however, the earlier studies did not determine the contribution of the tau oligomer reduction to the resulting neuroprotection observed with TIA1 reduction. The current studies clearly demonstrate that TIA1 reduction decreases the production of toxic tau oligomers.

The pattern of cytoplasmic localization of the propagated oligomeric tau was similar to that previously observed for TIA1 and neuronal SGs, suggesting the potential for intracellular interactions between propagated oligomeric tau (generated by templating of propagated tau), TIA1 and SGs. Indeed, binding of TIA1 to tau oligomers generated by templating of extracellularly propagated tau could promote their stabilization and accumulation [2]. This subcellular localization of cytoplasmic TIA1 might in turn reflect the roles of Tau, TIA1 and SGs in regulating ribosomes, which are most abundant in the soma [29, 43]. Thus, this study provides striking evidence of the pathophysiological importance of tau oligomers, indicates a role for TIA1 and associated SGs in the accumulation of propagated tau oligomers and provides a novel molecular mechanism controlling these processes.

The direct role of TIA1 in the mechanism of toxicity mediated by propagated tau was proven by showing that TIA1 reduction decreased degeneration associated with propagated tau. PS19::*Tia1*^+*/*−^ mice were highly resistant to neurodegeneration mediated by the S1p oligomeric fractions from PS19 mice, and S1p oligomeric fractions from PS19::*Tia1*^+*/*−^ mice exhibited reduced toxicity in cultures of hippocampal neuron over-expressing tau. Although the relative contributions to neurodegeneration from cell synthesized tau and intercellular propagated tau are not well understood for PS19 P301S tau mice aging normally, the current study used templated tau fractions to greatly accelerate production of tau pathology, which means that most of the tau pathology studied resulted from tau propagation. The neuroprotection provided by TIA1 reduction demonstrates its requirement in neurodegeneration mediated by propagated, templated tau oligomers. Thus, the current study demonstrates for the first time that TIA1 regulates neurodegeneration mediated by both intrinsic and extrinsic tau, with TIA1 regulating toxicity by determining the amount of toxic tau oligomers and the response of neurons to the toxic tau oligomers. The discovery that these neurodegenerative pathways share a unified mechanism mediated by tau oligomers, TIA1 and SG biology, suggests that therapeutic approaches targeting this pathological mechanism will be able to inhibit both the cell synthesized (intrinsic) and intercellular propagated tau pathways in tauopathy, leading to increased therapeutic efficacy. We suggest that inhibiting the interaction of TIA1 with tau might be one such approach.

## Electronic supplementary material

Below is the link to the electronic supplementary material.

Supplementary material 1 (DOCX 11731 kb)
