# Supplementary material for: TIA1 regulates the generation and response to toxic tau oligomers
Source: Acta Neuropathol. 2018 Nov 21;137(2):259–77. doi: 10.1007/s00401-018-1937-5 (PMC6377165; doi:10.1007/s00401-018-1937-5)
Supplement: Supplementary file 1 — Supplementary material 1 (DOCX 11731 kb) [file 401_2018_1937_MOESM1_ESM.docx]

**Supplemental Information:**

**TIA1 regulates the generation and response to toxic tau oligomers**

Lulu Jiang^1^, Peter E. A. Ash^1^, Brandon F. Maziuk^1^, Heather I. Ballance^1^, Samantha Boudeau^1^, Ali Al Abdullatif^1^, Marcello Orlando^1^, Leonard Petrucelli^2^, Tsuneya Ikezu^1,3^, Benjamin Wolozin^1,3*^

^1^Department of Pharmacology and Experimental Therapeutics, Boston University School of Medicine, Boston, MA, 02118.

^2^Neuroscience Division, Mayo Clinic, Jacksonville, FL 32224.

^3^Department of Neurology, Boston University School of Medicine, Boston, MA, 02118

* Correspondence should be addressed to Benjamin Wolozin, M.D., Ph.D.,
Depts. of Pharmacology and Neurology
Boston University
School of Medicine
72 East Concord St., R614
Boston, MA 02118-2526
617-414-2652 (Phone)
[bwolozin@bu.edu](mailto:bwolozin@bu.edu)


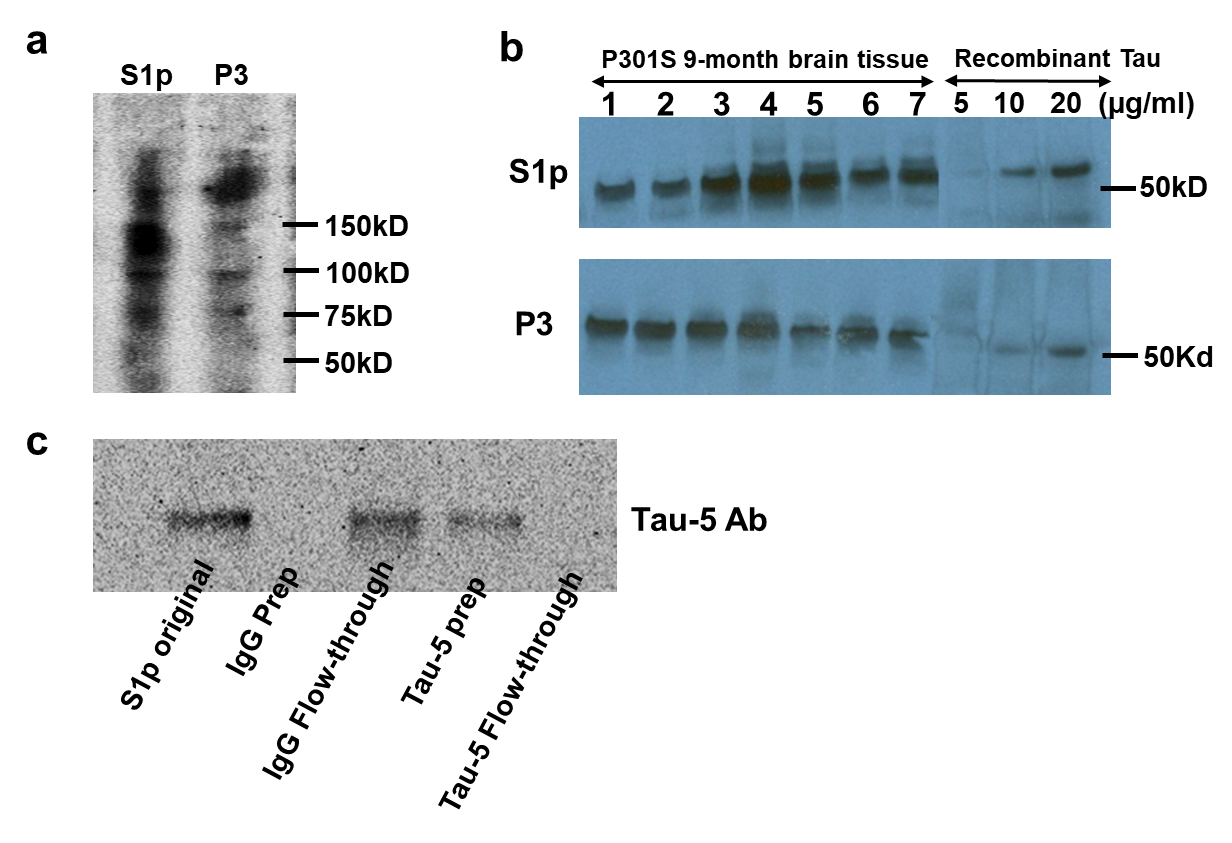


**Supplemental Fig. 1 (Related to Fig. 1). Quantification of S1p and P3 fractions. a** Western blot of S1p and P3 fractions extracted from 9-month old PS19 brain tissues. Native page gel electrophoresis and western blot by tau-5 antibody showed the molecular weight (M.W.) difference of Tau aggregates in S1p and P3 fractions. S1p fractions were enriched of tau dimers and trimers with M.W. between 100-150 kD while P3 were enriched of fibrils with M.W. more than 150 kD. **b** Bolt 4-12% reduced gel electrophoresis and western blot used for quantifying the total tau level of S1p and P3 with gradient recombinant tau ladders. The total human tau in S1p or P3 fractions are detected by tau13 antibody. S1p or P3 concentration were all normalized to 20ug/ml based on the concentration determined by western blot. That is, 2µl equal to 40ng. **c** Efficiency of tau oligomers absorption by immuno-precipitation was detected by tau-5 antibody western blot. It showed that tau-5 antibody efficiently deplete tau from S1p fractions while IgG had no influence for the total tau in S1p fractions.

**
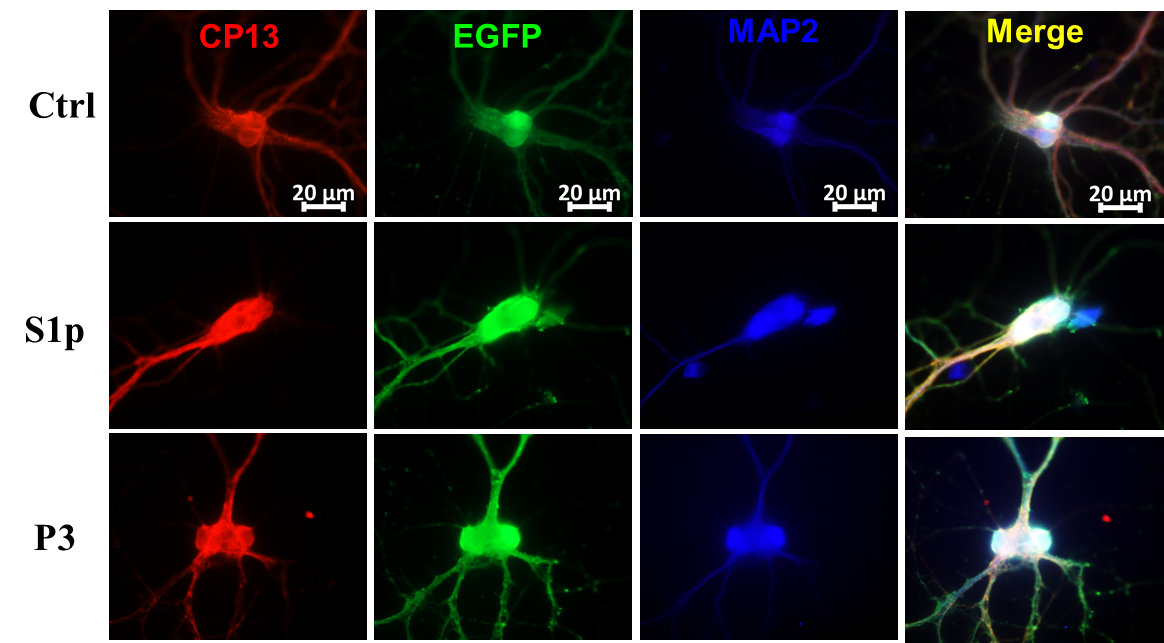
**

**Supplemental Fig. 2 (Related to Fig. 1). S1p or P3 didn’t induce significant tau inclusions in C57 wild type hippocampal neurons with EGFP over-expression by a AAV1 vector control.** Primary hippocampal neurons were transduced with EGFP-AAV1 vector control side by side to 4R0N WT tau or P301L tau AAV1 on DIV-2 of cell culture in 24-well plate. On DIV-14, neurons were treated with 2μl S1p or P3 and then fixed at 24 hours after treatment. Immuno-fluorescence staining was performed to detect tau inclusions induced by S1p or P3. The representative images here showed that S1p or P3 didn’t induce significant tau inclusions in C57 wild type hippocampal neurons, which was labeled by hyper-phosphorylation marker CP13 (red). And the limited tau inclusions in neurons were not co-localized with EGFP (green) in neurons (blue). Scale bar 20 μm.


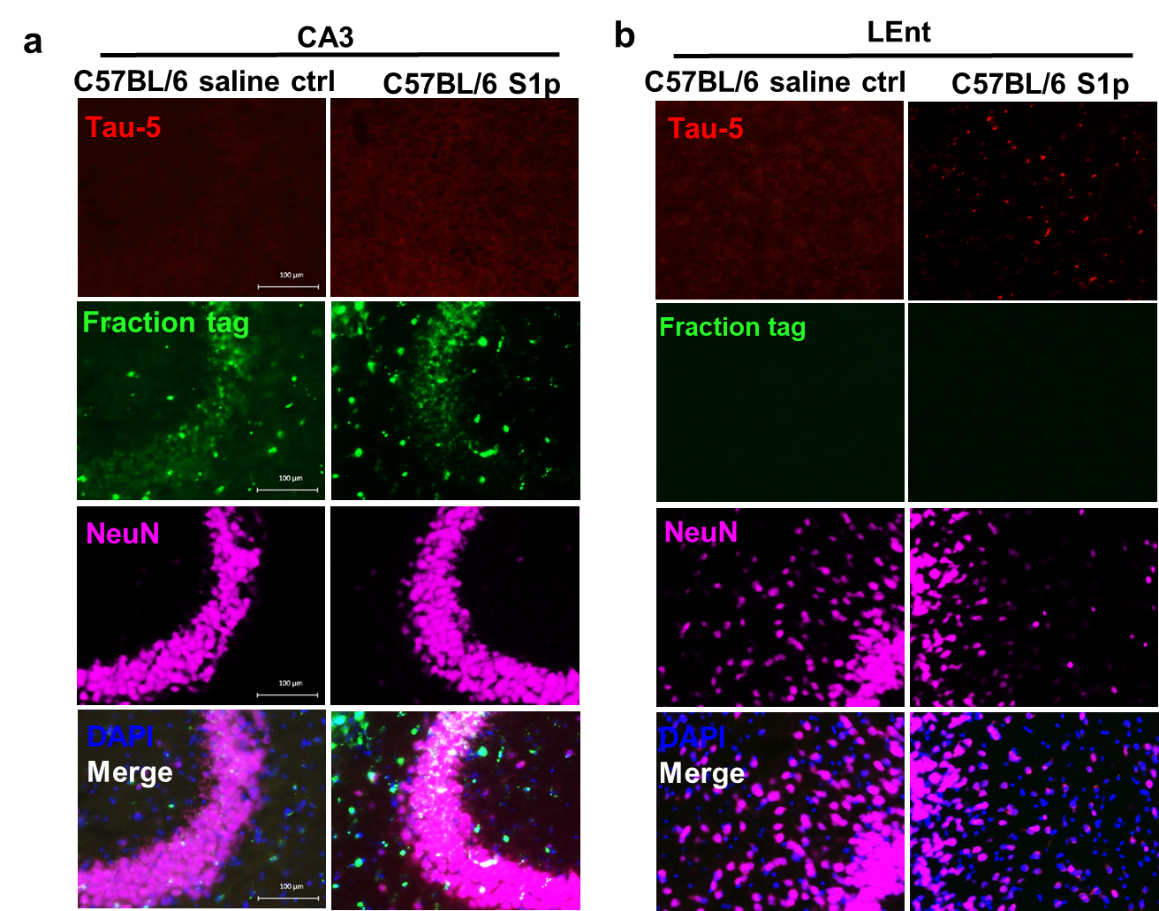


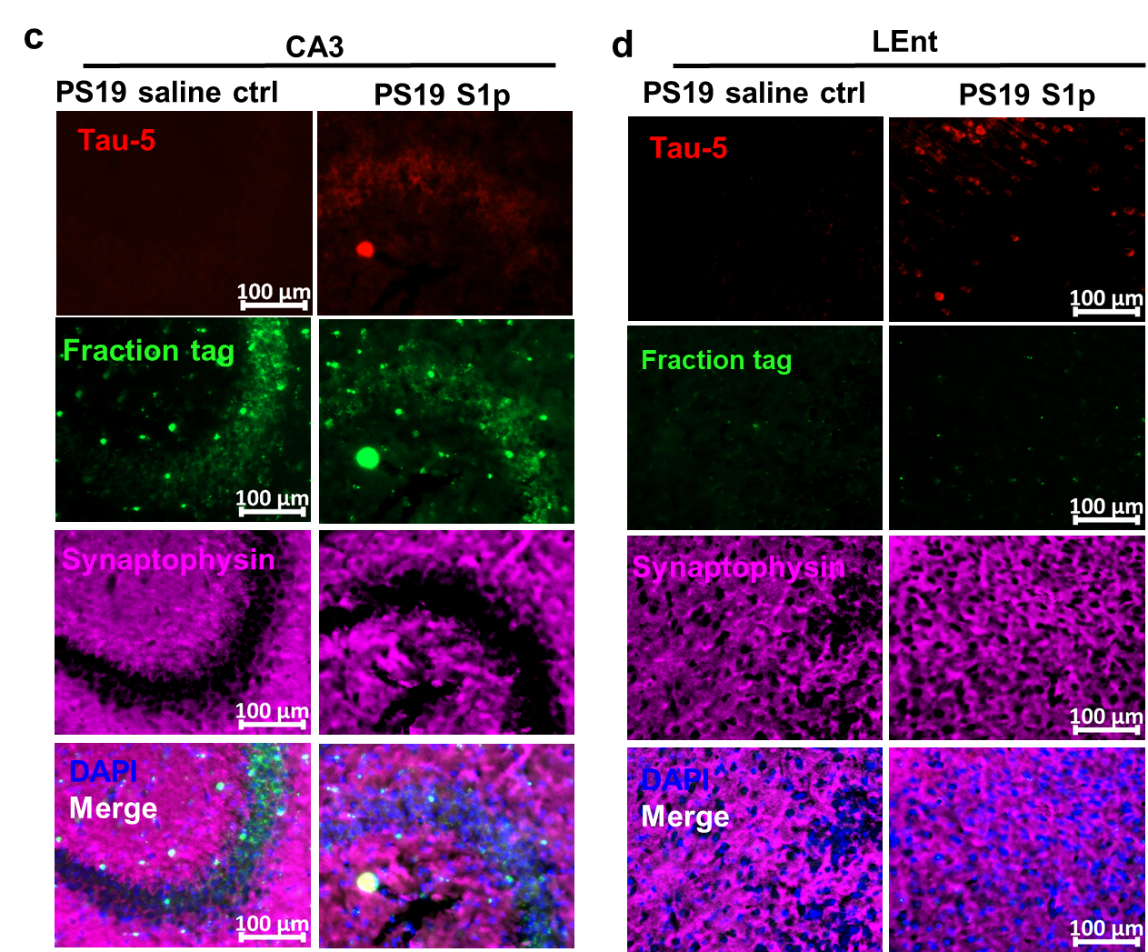


**Supplemental Fig. 3 (Related to Fig. 2). The injected S1p fractions containing tau aggregates could diffuse into CA3 in some degree but cannot diffuse into distal LEnt region.** To clarify the possibility that the increased tau inclusions in CA3 and LEnt were due to diffusion from injected CA1 brain region, saline or S1p fractions were covalently labeled with dylight 488 on the amines residues prior to injection (kit from abcam, cat#ab201799). The injected C57BL/6 wild type mice were sacrificed at 3 months after injection (litter mate of and same time line as to PS19 mice). Or the injected PS19 mice were sacrificed at 15 days after injection. **a** Representative images showed that the amount of dylight 488 (either in saline or tagged to tau) diffused to CA3 region at a level that was similar for the saline control and S1p fractions in WT type mice at 3 months after injection. **b** Tagged fractions were not found in LEnt brain region of WT mice after S1p injection for 3 months. Scale bar 100 µm. **c** Representative images showed that the tagged fraction also diffused to CA3 region to a similar degree between saline control and S1p fractions at 15 days after injection in PS19 mice. **d** Tagged fractions were not found in LEnt brain region. These results suggested that the increased tau inclusions triggered by S1p fraction at short or long terms were primarily from the templating of tau produced in the recipient neurons. Scale bar 100 µm.

**
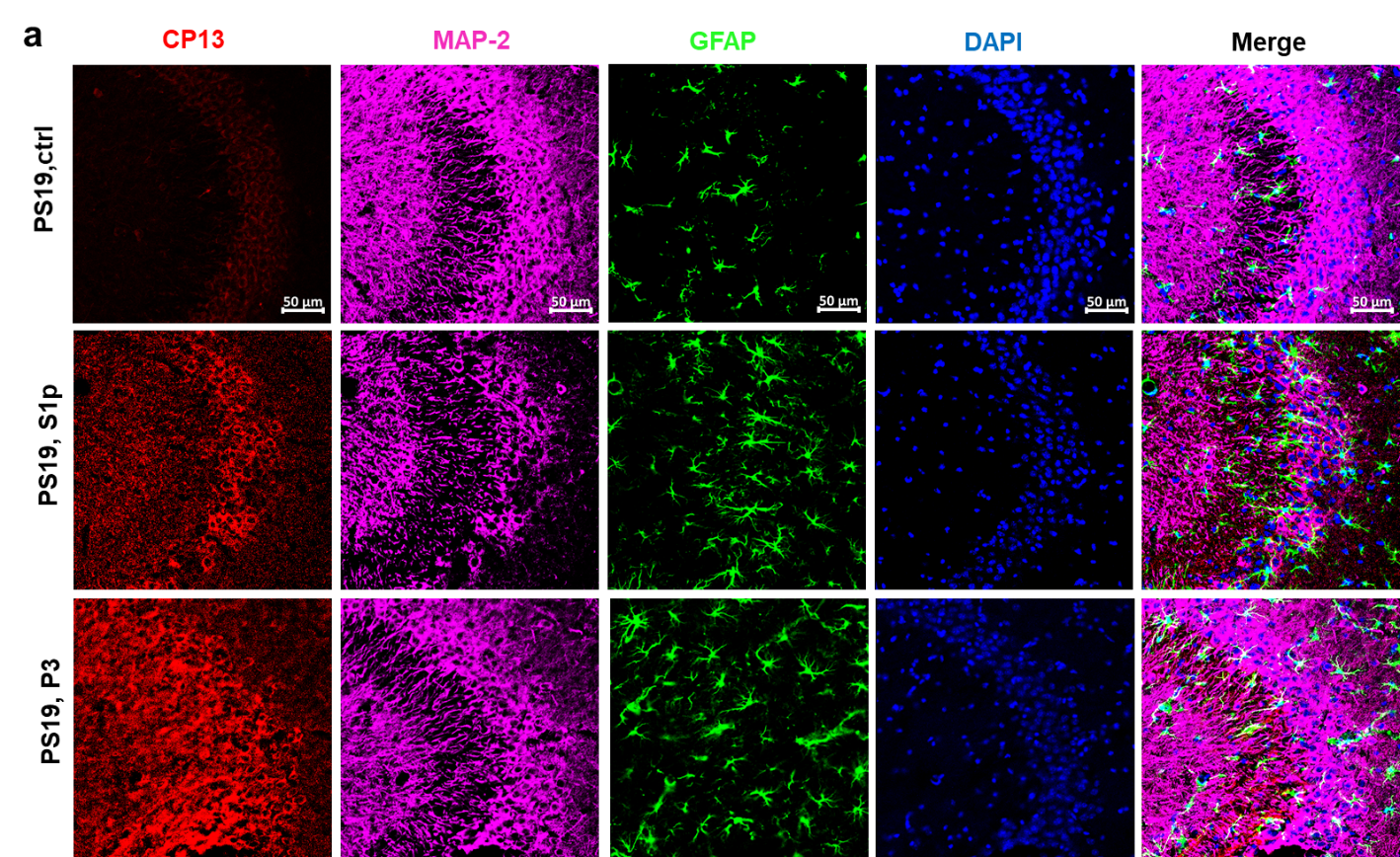
**

**
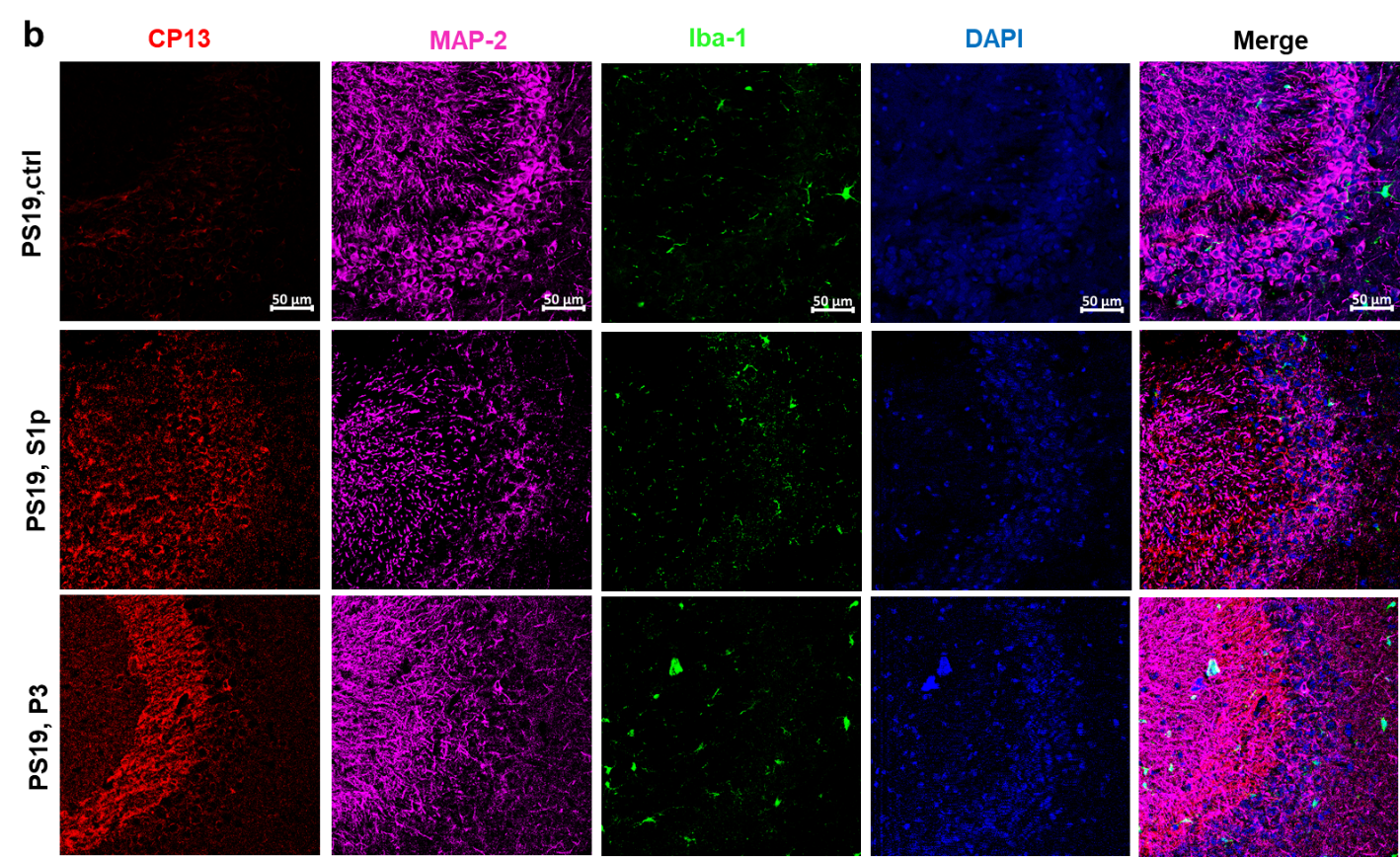
**

**Supplemental Fig. 4 (Related to Fig. 2). S1p or P3 induced tau propagation was primarily within neurons instead of glial cells.** After 3 months of S1p or P3 injection, the fixed PS19 brain sections of hippocampus were co-stained with CP13 (hyper-phosphorylated tau marker, red), MAP-2 (dendritic marker, violet), GFAP (marker for astrocyte, green in **a**) or Iba-1 (marker for microglia, green in **b**) to confirm the location of tauopathy in different cell types. The representative images here indicate that CP13 has minimal overlap with GFAP or Iba-1, suggesting that S1p or P3 induced tau propagation primarily in neurons. Scale bar 50 µm.


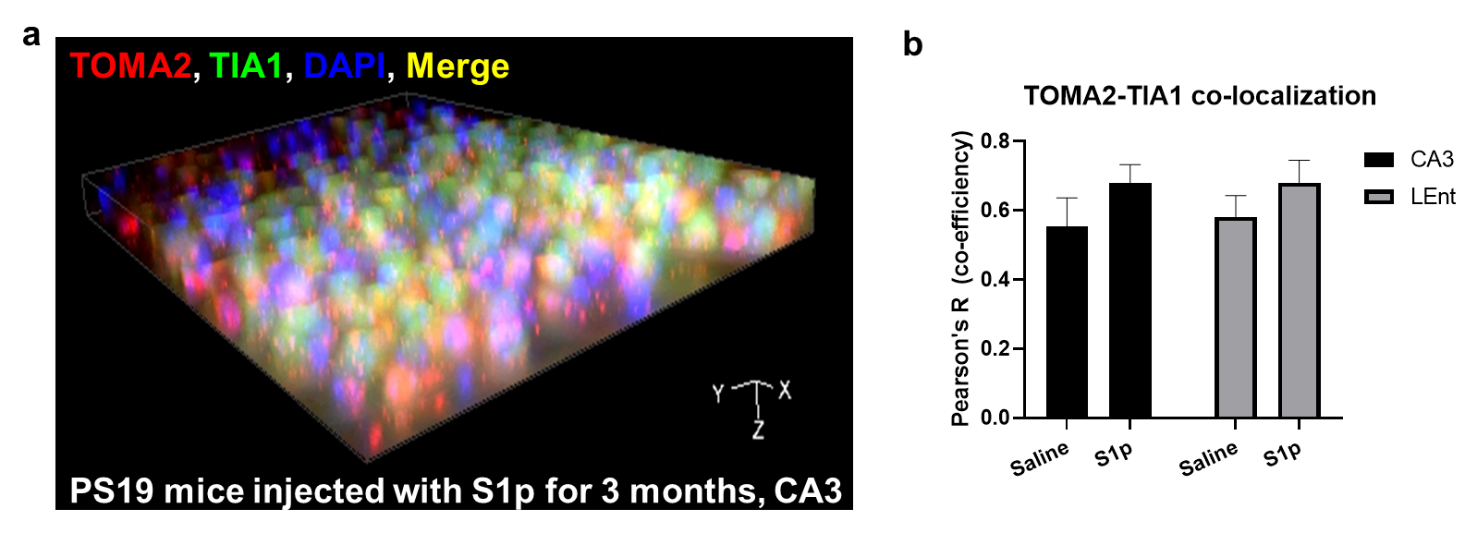


**Supplemental Fig. 5 (Related to Fig. 3). Three-dimension image showed the co-localization of TIA1-TOMA2 granules in neuronal cell body. a** The representative 3-dimension image compressed from z-stack imaging of PS19 CA3 brain sections after S1p injection showed that tau oligomers (TOMA2, red) c0-localized with TIA1 (green) granules in neuronal cell body. **b** co-efficiency analysis of co-localization of TIA1-TOMA2 in PS19 mice with S1p or saline control injection. Pearson’s R showed that oligomeric tau (labeled by TOMA2) and TIA1 have strong co-relation under basal condition as well as when with S1p propagation.

**
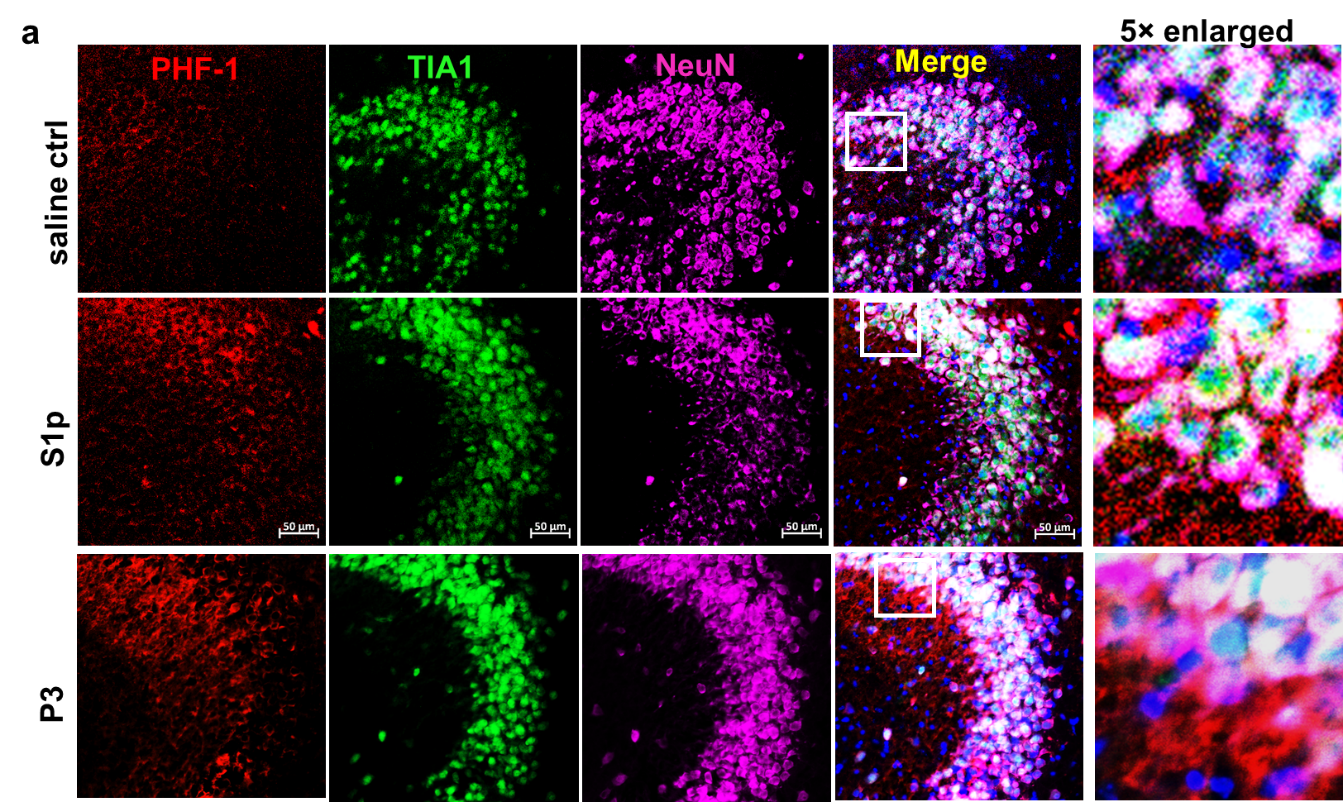
**

**
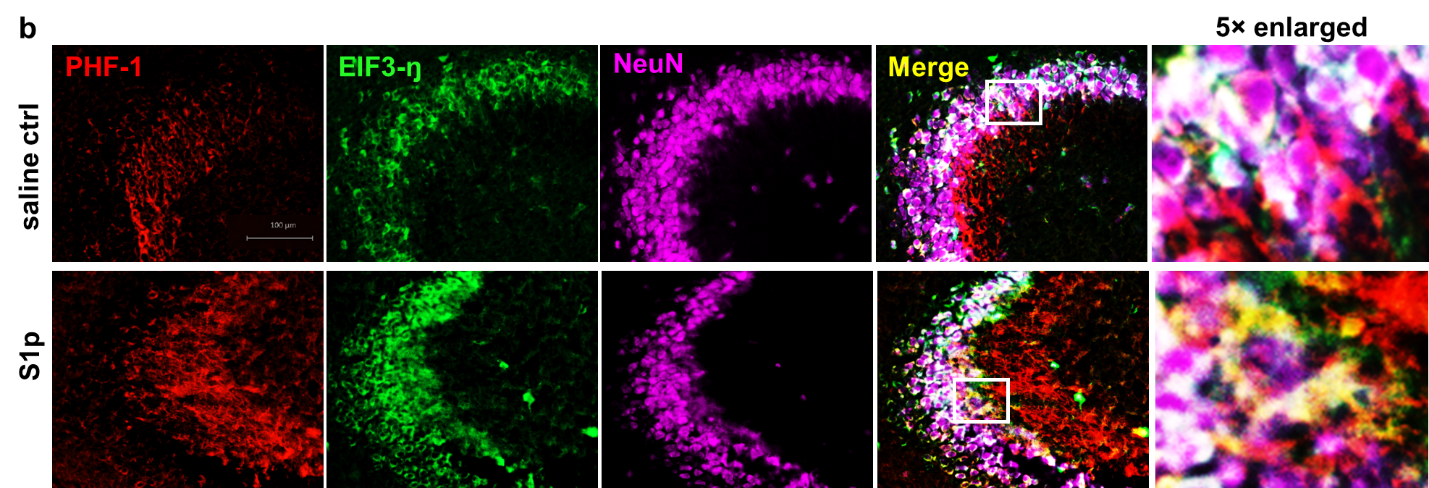
Supplemental Fig. 6 (Related to Fig. 4). Oligomeric tau spreading elevated phosphorylated tau aggregation and co-localization with RNA binding proteins. a** To confirm that S1p increased phosphorylated tau aggregation and co-localization with TIA1, we performed a co-staining of PHF-1 (another pTau marker besides CP13) with TIA1 of CA3 brain sections. It showed that both S1p and P3 fractions increased intensity of PHF-1 staining but only S1p induced tau inclusions were co-localized with TIA1 in soma. Scale bar 50 µm. **b** To investigate whether S1p elicited tau inclusions were co-localized with other RNA binding proteins, co-staining of PHF-1 and EIF3-ŋ (another RNA binding protein besides TIA1) were performed. Representative images showed that oligomeric tau spreading and co-localized with EIF3-ŋ in neuronal soma but not in processes. Scale bar 100 µm.

**
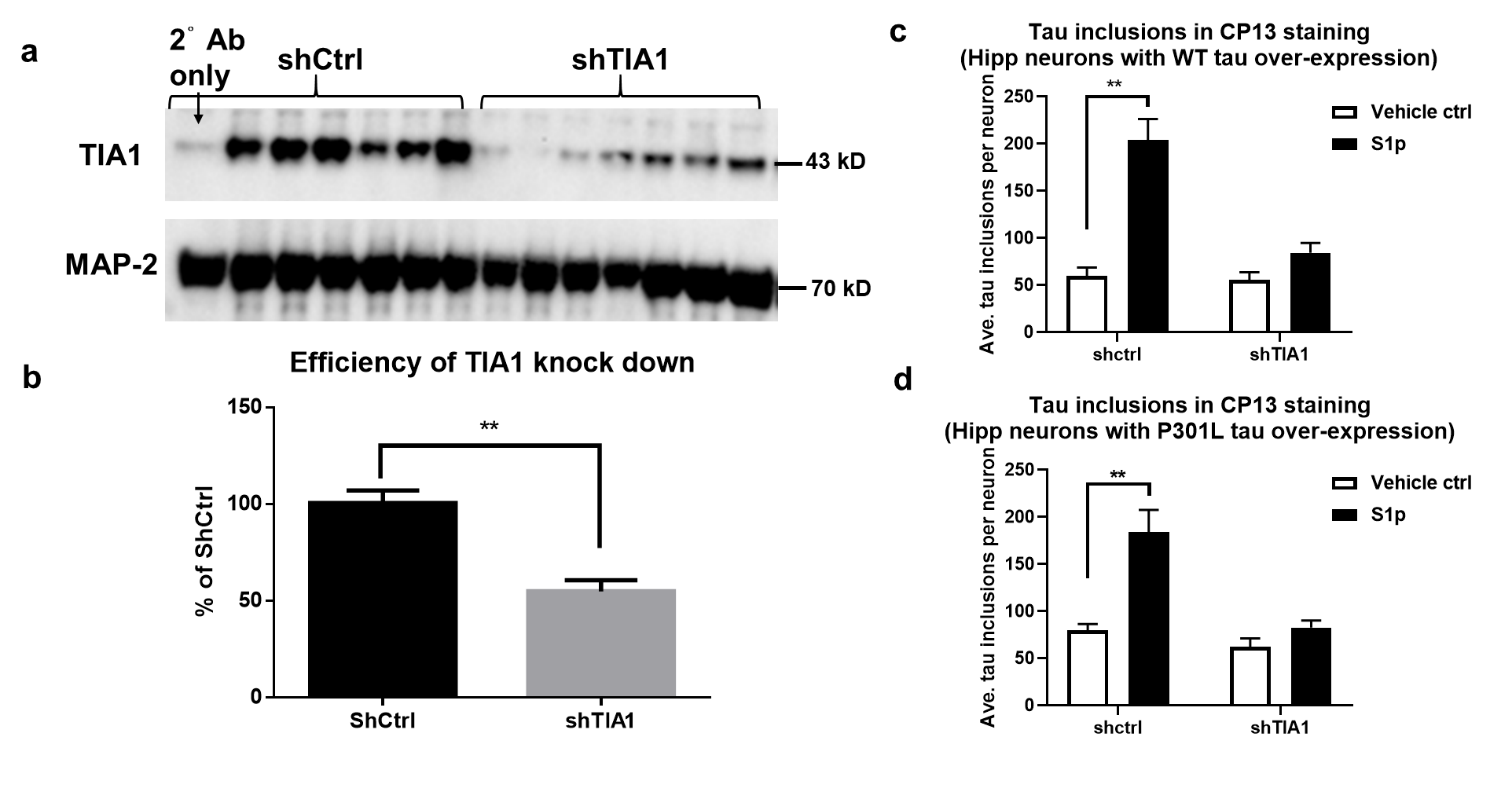
**

**Supplemental Fig. 7 (Related to Fig. 7). TIA1 knock down by shRNA showed more than 50% of TIA1 decrease in the cell lysate of hippocampal neurons.** Postnatal day-0 hippocampal neurons were over-expressed with human 4R0N WT or P301L Tau on DIV-2 with AAV1 and knocked down of TIA1 with AAV9 on day-4. The neurons were harvested on DIV-14 and cell lysate was collected for western blot. **a** Bolt 3-12% reducing gel western blot showed the amount of TIA1 in each sample lysate and it was normalized by neuronal marker MAP-2. **b** Quantification of western blot showed shTIA1 knock down reduced 45% of TIA1 in hippocampal cultures. ***p*=0.0004 by two-tail *t*-test, N=6, data expressed as mean ± SEM. **c-d** quantification of CP13 labeled tau inclusions after S1p administration in hippocampal neurons with WT 4R0N tau (c) or P301L tau (d) over-expression. The result showed that TIA1 knock down significantly reduced S1p induced tau aggregation.
